# Biobank-scale genotyping of Robertsonian translocations reveals hidden structural variation on the human acrocentric chromosomes

**DOI:** 10.64898/2026.03.08.710242

**Published:** 2026-03-27

**Authors:** Arang Rhie, Juhyun Kim, Francisco Rodriguez-Algarra, Steven J. Solar, Sergey Koren, Dmitry Antipov, Caralynn M. Wilczewski, George L. Maxwell, Jennifer Gerton, Justin Paschall, Tamara Potapova, Tyra G. Wolfsberg, Sumeeta Singh, Sandra O. del Castillo del Rio, Human Pangenome Reference Consortium, Clesson Turner, Vardhman K. Rakyan, Adam M. Phillippy

**Affiliations:** 1 Genome Informatics Section, Center for Genomics and Data Science Research, NHGRI, NIH, Bethesda, MD, USA; 2 The Blizard Institute, School of Medicine and Dentistry, Queen Mary University of London, London, UK; 3 Centre for Epigenetics, Queen Mary University of London, London, UK; 4 Reverse Phenotyping Core, Center for Precision Health Research, NHGRI, NIH, Bethesda, MD, USA; 5 Women’s Health Integrated Research Center, Inova Health System, Falls Church, VA, USA; 6 Stowers Institute for Medical Research, Kansas City, MO, USA; 7 Bioinformatics and Scientific Programming Core, Office of Scientific Core Facilities, NHGRI, NIH, Bethesda, MD, USA

**Keywords:** Balanced Robertsonian translocation, Distal junction of the rDNA, Structural variation, acrocentric chromosomes, telomere-to-telomere genomes

## Abstract

Balanced Robertsonian translocation (ROB) is the most common chromosomal rearrangement in humans, with an estimated occurrence of 1 in 800 in newborn studies. Carriers are at increased risk of cancer and often diagnosed at fertility clinics after facing recurrent miscarriages, infertility, or aneuploid offspring. Genotyping carriers with DNA sequencing has been challenging because of gaps and misrepresentation of the translocation fusion site in the human reference genome. Only recently, telomere-to-telomere (T2T) human genomes successfully revealed sequences of the acrocentric short arms, including the most common ROB fusion site. A ROB results in loss of two ribosomal DNA (rDNA) arrays and its adjacent distal sequences, including the highly conserved distal junction (DJ). Here, we present a novel method to type ROB carriers directly from short sequencing reads by estimating DJ copy number. We demonstrate that our method successfully genotypes ROBs using a reference-free approach or alignments to either T2T-CHM13v2 or GRCh38. Applying the method to a cohort of healthy newborns and family members (n=4,172) as well as the UK Biobank (n=490,416), we find candidate ROBs at a frequency consistent with the previously reported 1 in 800 incidence (0.11–0.12%). In addition to ROB carriers, we report the frequency of one DJ loss (9, 2.8–3.4%) or gain (11+, 8.4–9.3%) from the two cohorts and the 1000 Genomes Project (n=3,202), and characterize the underlying structural variation in near-T2T genome assemblies from the Human Pangenome Reference Consortium. Importantly, our method provides the first sequencing-based diagnostic for Robertsonian chromosomes and can be applied to low-coverage sequencing data, enhancing its clinical applicability and enabling new studies of structural variation on the acrocentric chromosomes.

## INTRODUCTION

Robertsonian translocation is a type of chromosomal fusion involving acrocentric or telocentric chromosomes. In humans, Robertsonian chromosomes (ROBs) occur at a rate of 1 in 800 individuals^1^, with the most common form of translocation found between chromosome 14 and either chromosome 13 or 21, accounting for 75% and 10% of cases, respectively^2,3^. If a person carries a ROB with two normal chromosomes to pair, it is considered a “balanced translocation” (hereafter shortened as “ROB carrier”) as opposed to monosomy or trisomy. For example, one fused ROB chromosome between 14 and 21 is still able to pair with the unaffected copies of 14 and 21 during meiosis. However, such carriers are at increased risk of childhood leukemia, non-Hodgkin lymphoma and breast cancer^4^. The carriers are also at increased risk to produce gametes with an unbalanced karyotype, often leading to spontaneous miscarriage, infertility, and uniparental disomy or trisomy in the offspring^5^. The extra 21q copy from an inherited ROB chromosome has been reported to cause 2–4% of Down syndrome cases^6^. Similarly but less frequently, an additional 13q copy can be transmitted from a ROB chromosome, causing Patau syndrome^7^.

The exact mechanism and breakpoint of the fusion site forming the most common type of ROB remained unknown until recently. In the past, ROB formation was classified as a centromeric fusion^8^ or referred to as a fusion of unknown microsatellites^9^. The first complete telomere-to-telomere (T2T) human genome revealed the complex structure of the acrocentric p-arms, including regions with high sequence homology^10^. In a follow-up study, using human pangenome assemblies^11^, frequent inter-chromosomal exchange was inferred within so-called pseudo-homolog regions (PHR)^12^. A particular PHR on the proximal short arms of Chrs. 13, 14, and 21 was suggested to mediate ROB translocations^13^, which was later confirmed by the T2T sequencing and assembly of three ROB cell lines^14^. Such ROB formation results in the loss of one fusion site and sequences distal to it, which includes the rDNA gene array on each chromosome and the highly conserved distal junction (DJ) sequence of each rDNA array^12,14,15^.

Until now, the most widely used method for identifying ROB carriers relies on karyotyping, fluorescence in situ hybridization (FISH)^16^ or Hi-C^17^. However, these methods depend on live cells or intact tissues and are labor intensive. Thus, these methods may not be applicable for routine diagnostics or large-scale cohorts. Microarray or SNP based chips have also been developed to detect ROB carriers^18^. However, the lack of understanding of the exact ROB breakpoint and gaps in the prior human reference genomes (i.e. both GRCh38 and hg19) have posed a barrier to using whole genome sequencing (WGS) reads to detect ROB carriers^19^.

Recognizing that ROBs result in the predictable loss of acrocentric sequence along with the increasing large-scale cohorts sequenced with WGS available, we sought to explore methods to identify candidate ROB carriers from such datasets. Here, we show three ways to detect ROBs from short-read sequencing data by using (1) T2T-CHM13/hs1 as an alternate reference for read alignment, (2) pre-existing read alignments mapped to GRCh38, and (3) DJ marker *k*-mers and their multiplicity directly obtained from sequencing reads. We benchmarked these methods to reference genomes CHM13 and HG002 as controls and three known ROB cell lines as cases^10,14^. Next, we applied the method on WGS in the Reverse Phenotyping Core (RPC) cohort (n=4,172)^20,21^ and the UK Biobank WGS cohort (n=490,444)^22^. We report that these methods identify potential ROB carriers in all cohorts at approximately the expected frequency of 1 in 800. Finally, we typed all samples from the 1000 Genomes Project (1KGP)^23^ and further examined samples with atypical copies of the DJ in the recent, near-T2T HPRC Release 2 assemblies^24^. We propose this method as a routine screening approach for identifying potential ROB carriers in any whole-genome sequencing dataset.

## RESULTS

### Detecting ROBs by the loss of rDNA distal junctions

**Fig. 1 |:**
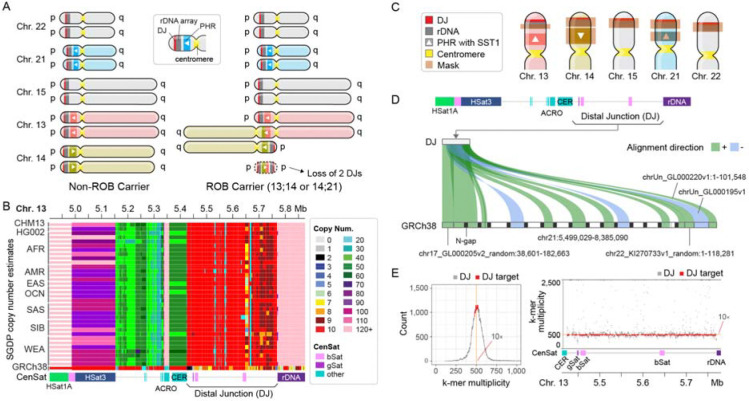
Target rDNA distal junction (DJ) for ROB carrier detection. (A) Schematic overview of the most common karyotype of ROB carriers. Chr. 14 pseudo-homolog region (PHR) has been identified in its inverted orientation compared to those on Chrs. 13 and 21, reported as the fusion site for the most common forms of ROBs. When a ROB is formed through a fusion between two acrocentric chromosomes, e.g. Chrs. 14 (lime) and 13 (red), one small segment carrying one PHR and two DJs gets lost. Legends follow as in panel C. (B) Copy number estimates of the DJ shows conserved 10–20 copies across individuals from various ancestral backgrounds in the Simons Genome Diversity Project. No other suitable regions on the short arms were found with a stable copy number. Copy numbers for GRCh38 (at bottom) indicate this region is mis-assembled in GRCh38. SGDP population labels: AFR, Africa; AMR, America; EAS, East Asia; OCN, Oceania; SAS, South Asia; SIB, Siberia; WEA, Western Eurasia. (C) Masked regions of the T2T-CHM13v2.0 acrocentric p-arms for copy number estimation of the DJ, rDNA, and PHR. Only one copy of each in Chr. 13 has been chosen as the target for copy number estimates. (D) Identifying DJ target region for copy number estimates for sequencing read alignments on GRCh38. Nine mis-assembled copies of the target were found on Chr. 21 surrounded by gaps, two copies on unplaced contigs and two on unlocalized contigs of Chrs. 17 and 22. (E) Reference free DJ copy number estimates. A *k*-mer found in all five DJs in T2T-CHM13v2.0 were selected as “DJ *k*-mers”. Based on the *k*-mer copy number count in Illumina reads (left), *k*-mers with ~10× coverage were selected as “DJ target *k*-mers”. The *k*-mer multiplicity on CHM13 Chr. 13 shows a stable 10× coverage across the region for the DJ target *k*-mers.

We first identified the most stable, highly conserved region to target for copy number estimates. The most common form of ROB translocation is reported to have a breakpoint in the proximal short arm, between the rDNA and centromere^14^. Therefore, ROB carriers are expected to be missing two rDNA arrays and all sequences distal to these arrays (Fig. 1A). Because of the difficulty in detecting the loss of rDNA units due to their highly variable copy number, we targeted the region immediately distal of the rDNA array, the DJ, which had been reported to be highly conserved in humans and expected to be in a single copy per chromosome^15^. Surveying the copy number estimates from the Simons Genome Diversity Project^10,25^, we confirmed that most of the DJ region maintains a stable 10 copy pattern across individuals regardless of ancestral background, suggesting the presence of a highly conserved, single DJ per rDNA array (Fig. 1B). Thus, we hypothesized that two DJ copies will be lost following most Robertsonian translocations, resulting in eight DJs in the ROB carriers. Using this hypothesis, we developed a pipeline for read-mapping based copy number estimates utilizing a masked T2T-CHM13 reference (Fig. 1C). Specifically, we masked all but one copy of the DJ in the T2T-CHM13 reference and compared the coverage against the background coverage collected from the rest of the non-heterochromatic autosomal regions. In addition, we masked all but one copy of the 45S rDNAs and Robertsonian-associated PHRs to estimate copy number for these regions as well (Table S1 and Fig. S1-4).

To expand the pipeline on large-scale cohorts already mapped to GRCh38, we sought to look for the presence of the target DJ sequence in GRCh38. When mapping GRCh38 to each target sequence extracted from CHM13, we identified nine DJ copies on GRCh38 chr21 organized as a repeat array with some inverted and incomplete copies interspersed and surrounded with gaps (Fig. 1D). This is an arrangement seen in no T2T-assembled genome since, and represents a clear mis-assembly in GRCh38. In addition, partial DJ sequences were found on two unplaced sequences (chrUn_GL000195v1 and chrUn_GL000220v1) and two unlocalized sequences (chr22_KI270733v1_random and chr17_GL000205v2_random), with high sequence identity (average 96.2%) (Tables S2-3). Diverged pieces of DJs were found on Chrs. 4 and 10, similar to those observed in CHM13, however these were excluded from targeting due to their low sequence identity (<83.4%) and low coverage (<2.8%).

In addition to the mapping-based method, we also developed a reference-free approach to reduce any potential mapping bias. To select target *k*-mers for the DJ, all *k*-mers (*k*=31) in T2T-CHM13v2 were evaluated for their copy number multiplicity observed in Illumina reads derived from CHM13. Among the *k*-mers found at least once in each of the five DJs (Chrs. 13, 14, 15, 21 and 22), target *k*-mers were selected from the ~10 copy peak to exclude highly repetitive or variable regions (Fig. 1E). To estimate the copy number of the DJs for each new sample, all *k*-mers are collected directly from the FASTQ files. The two-copy peak is then extracted from the entire *k*-mer histogram and compared to the median multiplicity of the target DJ *k*-mers. Since this method operates directly on FASTQ files, it eliminates the need for a mapping process, thus reducing computational resource requirements.

**Fig. 2 |:**
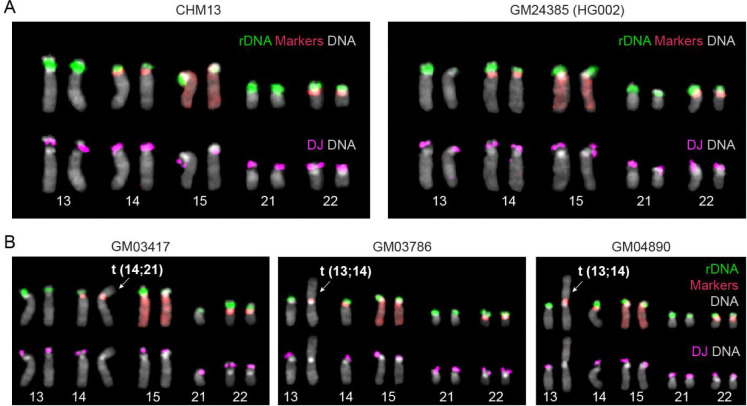
FISH karyotyping of the control cell lines. (A) Control cell lines CHM13 and GM24385 (HG002). All 10 acrocentric haplotypes show the presence of rDNA (green) and DJ (magenta). (B) Previously reported ROB cell lines, which the breakpoint has been identified as the PHR in de Lima *et al*.^14^ The ROB chromosomes are highlighted, showing the absence of the DJ.

To test the pipelines, we used Illumina reads from CHM13, HG002 (GM24385)—for which 10 DJs were confirmed^10,26^—and ROB cell lines GM03417, GM03786, and GM04890—for which 8 DJs and ROBs were confirmed by T2T assemblies and FISH karyotyping by de Lima *et al.*^14^ (Fig. 2). We applied our mapping-based method using both GRCh38 and CHM13 reference genomes, utilizing BAM files aligned to each reference, as well as our *k*-mer-based method using raw sequencing reads. All three methods consistently reported ~10 DJs for both CHM13 and HG002 and ~8 DJs for the 3 known ROB cell lines, as expected (Table 1, Fig. 2 and Fig. S5).

### One in 800 ROB candidates in newborn family cohort

**Fig. 3 |:**
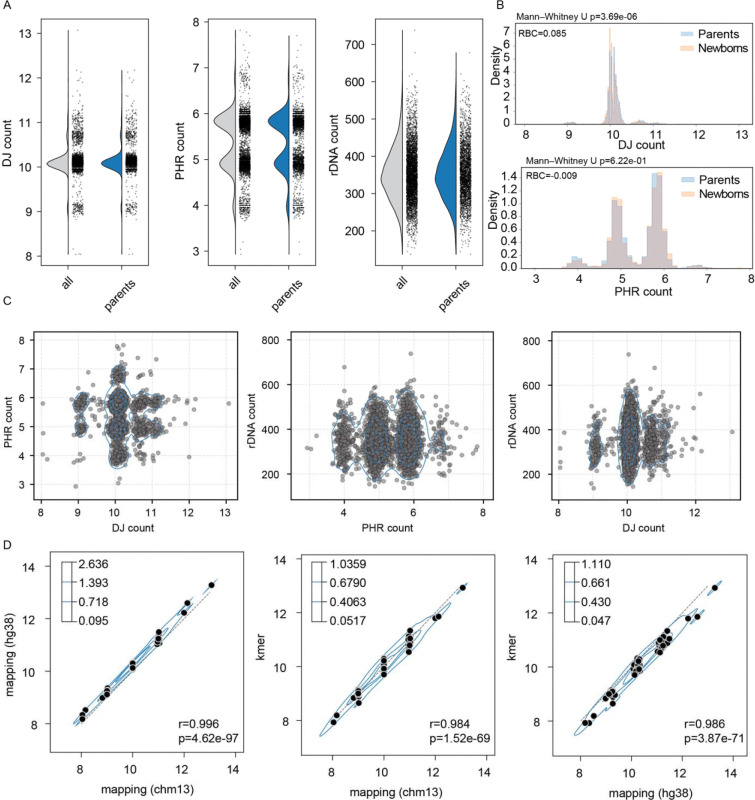
DJ, PHR, and rDNA copy number estimation using T2T-CHM13, GRCh38, and *k*-mer based methods for the RPC cohort. (A) Diploid copy number estimation of DJ, PHR, and rDNA in the RPC cohort using CHM13-based mapping approach. (B) Histogram of the probability density of DJ and PHR copy numbers across samples, stratified by parents and newborns. The area under each group’s histogram sums to one. A two-sided Mann–Whitney U test was performed using raw counts to compare parents and newborns, and the rank-biserial correlation (RBC) is reported as the effect size. (C) Each point represents a sample from the RPC cohort. No obvious correlations were observed between the DJ, PHR, and rDNA copy numbers. (D) Comparison between DJ counts derived from mapping-based estimates (CHM13 and GRCh38) and the *k*-mer–based method using subsampled samples (n = 92), showing strong correlation. Density levels are shown with four contour levels.

The Reverse Phenotyping Core (RPC) cohort includes 1,325 families with healthy newborns, parents, and siblings (where available), totalling 4,187 samples. Blood samples were collected at the time of birth. Illumina short read data aligned to hg19 from Wilczewski *et al.*^21^ and Bodian *et al.*^20^ were extracted and re-aligned to the masked version of T2T-CHM13v2.0 and evaluated for the copy number of the DJs, PHRs and rDNAs (Fig. 3). In total, we found 4 individuals with estimated 8 copies of the DJs (Fig. 3A). This includes two parents and one mother-newborn (daughter) pair. (Table 2). Counting the 3 out of 2,650 presumably unrelated parents, among which 2,612 were sequenced, yields an estimated ratio of 1 in 871–883 (0.11%), matching closely the expected 1 in 800 (0.125%) incidence^1^. Among the 2,612 parents, 2,277 (87.17%) appeared as having 10 estimated copies, comprising the majority of the individuals (Fig. 3A). Interestingly, regardless of with or without the offspring, we found 3.34–3.45% individuals with 9 DJs, and 9.26–9.27% with more than 10 DJs, with 13 being the most copies observed (Fig. 3 and Table 3). When comparing the number of samples classified in each DJ copy bin, no significant difference was observed between XX and XY individuals (χ^2^ test of independence, *p*>0.5). We observed a few more 8 DJs in XX carriers (3 vs. 1), with one passed down from the mother to daughter, matching previous reports that ROBs are more frequently observed in XX samples and transmitted to XX offspring^27^. When comparing the distribution of DJ copy number between parents and offspring, we observed a slight shift toward higher copy numbers in the parental genomes (Mann–Whitney U test, p = 3.7 × 10^−6^; Fig. 3B and Fig. S6).

However, when comparing the number of PHRs, we observed a broader polymorphic copy number distribution compared to the DJs (Fig. 3A). Initially, we expected that the majority of individuals would carry six copies of the PHRs as observed in CHM13 and HG002, and five copies for those with 8 DJs due to the ROB as reported in Guarracino *et al*.^12^ and de Lima *et al.*^14^. A little over half the samples were observed as 6 copies (52.3% in all and 56.6% among parents), still forming the largest frequency, followed by 5, 4 and 7 copies (39.3–35.6%, 6.2–5.5% and 2.0–2.3%, respectively for all and among parents (Table 4). Among the parental genomes, the portion of 6 PHR copies observed was slightly higher in samples carrying XX over XY (57.9% vs. 55.2%). For 5 and 4 copies, however, the observed frequency was slightly higher in XY carriers over XX (36.5% vs. 34.7% and 5.8% vs. 5.1%). Unlike the DJ, the distribution of PHR copy numbers was not significantly different between parents and their offsprings (Mann–Whitney U test, *p*>0.5. Fig. 3B). The copy number of the PHRs in the 8 DJ samples also varied from 4 to 6 (Table 2). Distribution of the PHR copy number also showed no significant relationship with the DJ gains or losses and showed a very weak correlation when restricted to samples with 8 to 10 DJ copies (Pearson correlation, *r*=0.01, *p*=0.39), indicating there could be other polymorphic variations of the PHRs that are yet to be discovered (Fig. 3C).

Next, we examined the changes in rDNA copy number associated with DJ loss or gain. The rDNA copy number varied widely among samples, ranging from 137 to 738 copies (Fig. 3A). Unlike the DJ or PHR distribution, we observed a unimodal peak with a wide deviation (mean=352, sd=70.2) as previously reported for the UK Biobank cohort^28^. Again, the rDNA copy number distribution showed a very weak positive correlation for samples with 8 to 10 DJ copies (DJ < 10.4; Pearson correlation: r = 0.09, p = 8.1 × 10 ). However, for samples with 10 or more copies, copy number ranges were similar and less correlated (DJ >= 10.4, Pearson r = 0.12, p = 1.4 × 10^2^). (Fig. 3C). No significant correlation was observed between PHR copy number and rDNA copy numbers (p > 0.05) (Fig. 3C).

We then evaluated whether our two alternative approaches—using GRCh38 as the reference or using marker *k*-mers—yielded high correlation with the CHM13 mapping–based approach. We selected samples representing each classified DJ copy number category, including all samples with 8 DJ copies and all samples with more than 12 DJ copies (n = 92 total). Among the GRCh38-based modes, we selected the “Fast-precise” mode (more details in “Computing resources and prediction model” section and Methods), which showed the highest correlation with CHM13-based estimates and the lowest residual sum of squares (RSS) when fitting DJ copy numbers derived from GRCh38 alignments (Fig. S7 and Table S4). All three approaches showed similar distributions and strong correlations (Pearson correlation r² and p-values shown in Fig. 3D), demonstrating the robustness and broad applicability of these methods across different data types.

### DJ and PHR copy number distribution in UK Biobank

**Fig. 4 |:**
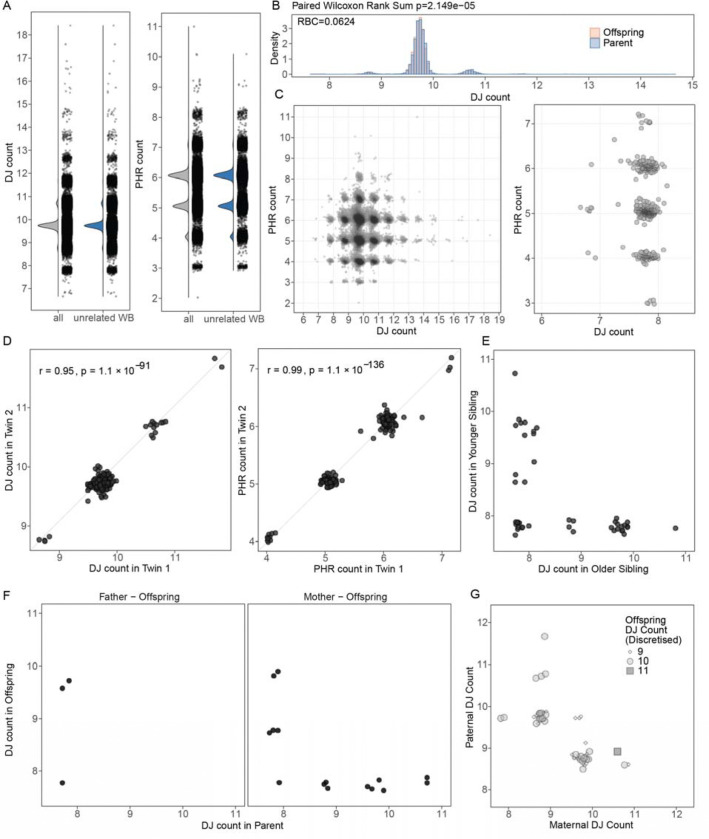
DJ and PHR counts in the UK Biobank. (A) DJ and PHR count distribution among all and unrelated white British (WB) samples. (B) Density distribution between the parent group and the offspring group. The RBC was calculated using the paired Wilcoxon Rank Sum test. (C) DJ copies as a function for PHR copy numbers. Left, for all DJs. Right, inset of the samples classified as 8 or lower. (D) DJ (left) and PHR (right) counts in monozygotic twins. The twins share the same copy number (Pearson’s r test). (E) DJ counts in siblings (except monozygotic twins) with one of the siblings determined to have 8 DJs. No particular inheritance patterns were found. (F) Inheritance pattern of all 8 DJ carriers found with father-offspring or mother-offspring relationships. No 8 DJ offspring with both parents were found in the cohort. (G) Inheritance pattern of the DJ in offspring among the available trios, with at least one member of the trio having 9 or fewer estimated DJs. No trio with 9 DJs in both parents was observed.

To confirm the distribution of the DJ copy numbers and the PHRs in an independent cohort, we applied our analysis to the UK Biobank (n=490,444), for which CRAM files aligned to GRCh38 are available (Byrska-Bishop *et al.*, Methods^23^). To enable DJ and PHR copy number estimation in this large cohort, we applied the mapping-based approach of our pipeline to reduce computational demands (“pilot Fast mode” in Methods and Fig. S8). Among the samples, we were able to calculate DJ and PHR counts for 490,369 and 490,374 samples, respectively. The distribution pattern and percent samples assigned to each discrete DJ and PHR count replicated what we observed in the RPC cohort, both when considering all individuals and when restricted to unrelated individuals (Fig. 4A). In total, we identified 583 samples with 8 DJ copies, corresponding to a predicted ROB frequency of 1 in 841 (0.12%), again closely matching the expected 1 in 800. Unlike the RPC cohort, we observed some individuals with 7 DJ counts. More individuals with 3 PHR counts were observed, presumably due to the larger size of the cohort, with a frequency of 0.05% regardless of the sex chromosome composition, and the lowest PHR count observed 2, in a single sample. Again, we did not observe a statistically significant difference in the frequency of each discrete copy number bin between sex (χ^2^ test of independence, *p*>0.5, Table 3). Unlike seen in the RPC cohort, we did not observe an increased number of 8 DJ carriers in XX individuals. We also confirmed that DJ counts were significantly (albeit slightly) higher not only in the parent group compared to the offspring (Fig. 4B), but also in older groups compared to younger groups (Fig. S9).

With the increased number of samples carrying 8 DJ copies, we were able to more precisely evaluate the relationship between PHR and DJ copy number. The overall distribution mirrored that observed in the RPC cohort, with a broader PHR copy number distribution among individuals with 10 DJ copies, again presumably due to the larger sample size (Fig. 4C). A higher proportion of individuals with 8 or fewer DJ copies had 5 PHR copies, as we initially hypothesized (54.4%, compared with 37.8% in individuals with 9 or more DJs). However, substantial individuals carried 3, 4, 6 or 7 copies of the PHRs (45.6%), suggesting other structural variations may be present altering the PHR counts. For example, prior HPRC assemblies showed 34.5% of the Chr. 14 haplotypes lacked the PHR (n=9 out of 26 haplotypes; de Lima *et al.*^14^, Extended Data Fig. 9], supporting our observation of variable PHR copy numbers in the population.

We further confirmed that monozygotic twins have identical DJ and PHR copy numbers (Fig. 4D), whereas dizygotic twins or siblings show differences in DJ copy number inheritance pattern (Fig. 4E), confirming the precision of our method. Among samples carrying 8 DJs, 9 samples had either the maternal or paternal genome available. Among the 3 paternal samples with 8 DJs, we found 1 case where the offspring inherited the same pattern (Fig. 4F left). Among the 6 maternal samples, 1 offspring had inherited 8 DJs (Fig. 4F right). The copy number of the offspring matched one of the parents in most cases, or were at 10 copies in most cases when one of the parents had ≥11 copies (Fig. 4G and Table S5). Three samples were found to have 9 DJs while both parental genomes had 10 DJs, suggesting a possible *de novo* loss of the DJ in the offspring. No cases were observed in which both parents had 9 DJs among the available trios.

### Uncovering structural variation within the DJ of near-T2T genomes

For detailed inspection of structural gains or losses in DJ copy number, we analyzed 1KGP samples both with and without near-T2T assemblies generated by the HPRC. The 1KGP samples have recently been re-sequenced using Illumina short reads (30×, n=3,202 among which n=2,504 are unrelated)^23^. A subset of the samples (n=226) have been long-read sequenced by the HPRC to build a reference pangenome from *de novo* assembled haplotypes. The cell lines were selected to broadly sample global genomic variation and were restricted to lines with a low passage count and normal karyotype to avoid artifacts from cell culture. These lines were assembled with Verkko^29^ using long reads, Hi-C and parental data when available^24^.

**Fig. 5 |:**
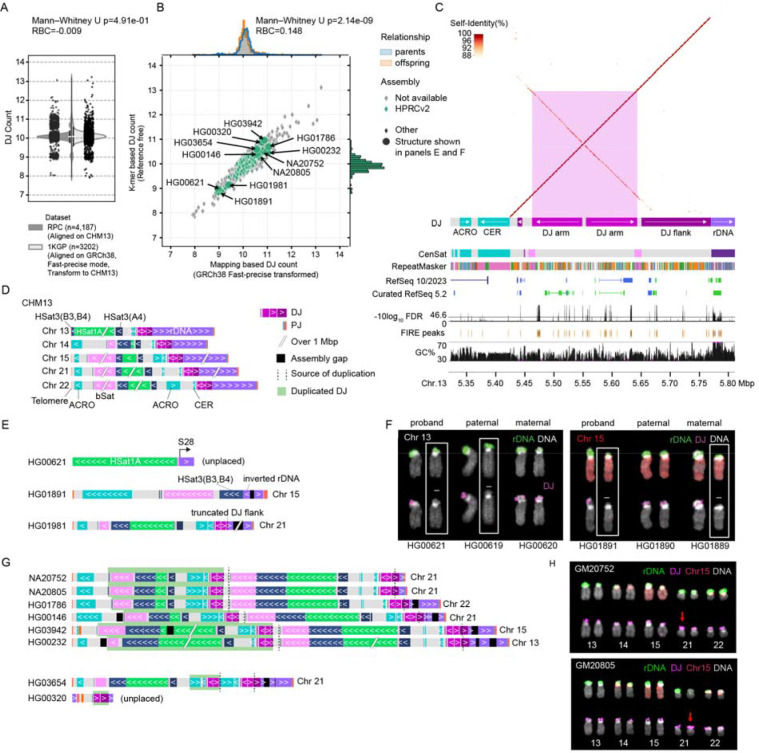
Polymorphism of the rDNA DJs and surrounding sequence on the short arms of the acrocentric chromosomes. (A) Distribution of DJ copy numbers in the RPC cohort, calculated using masked CHM13 as the ground truth, and in the 1KGP cohort, transformed from GRCh38 alignments (Fast-precise mode). (B) Distribution of DJ copy numbers across 1KGP samples (n = 3,202), with samples assembled by HPRC highlighted in orange. The normalized density plot (top) shows a slight shift of parental samples toward higher values among those with ≥10 copies. The right marginal plot displays the distribution of samples with assemblies. (C) Structure of the DJ and its functional components. The self-dotplot on top shows the palindromic arms inside the DJ, marked as DJ arm. The region flanking to the rDNA (DJ flank) shows a relatively unique pattern, with the beginning showing diverged inverted repeat shared at the beginning of the DJ (next to the CER). (D) Distal sequences and repeats as observed in CHM13. All DJ sequences are intact. Chr. 13 does not have the sequence below the bSat unlike the other sequences. (E) Ideograms of cases with loss of DJs. HG00621 had an unplaced sequence containing the loss of DJ. Sequences lost include the beginning of the rDNA array down to the bSat, resulting in loss of sequences including the entire DJ, CER, ACRO and HSat3(A4). The rDNA array started at the large subunit (S28). HG01891 lost sequences including the HSat1A, with a rare rDNA inversion found at the beginning of the rDNA array. HG01981 had only a partial loss of the DJ flank. Sequences larger than 1 Mbp are reduced and re-scaled for clarity. (F) FISH with probes for the DJs and rDNAs on the 2 cases found to have 9 DJs. HG00621 exhibited the same DJ loss pattern as the paternal genome, while HG01891 shared the pattern observed in the maternal genome. (G) Ideograms of cases with gain of DJs. Six samples shared the same duplicated form of DJ, sharing the same breakpoint at the DJ flank. The HSat1A block size varied, however the overall structure of the duplicated copy (green box) remained identical. The 2 samples at the bottom showed a unique structure, with one having the DJ palindromes duplicated and the inverted repeat part of the DJ flank. The last case shows a sequence within the rDNA array, with one rDNA array (left) having the PJ intact. This time, the additional DJ has only one of the DJ arms present with the DJ flank. (H) FISH for the two samples with 11 DJs. Because the DJs are too close to each other, and highly packed inside the heterochromatin, the duplicated DJs aren’t clearly distinguishable. Still, one haplotype (red arrow) exhibits stronger intensity compared to the other. The other haplotype of the same chromosome was assembled with a single DJ flanking the rDNA array.

We downloaded the Illumina BAM files aligned to GRCh38 from IGSR^30^ and counted the DJ copy numbers using the GRCh38 and *k*-mer based methods. To assess compatibility between methods and across independent cohorts, we applied previously derived linear models for each GRCh38 mode, using subsampled RPC data to transform DJ counts obtained from GRCh38 alignment into the CHM13-based distribution. This model was subsequently used to transform DJ counts in an independent 1KGP cohort. Among the evaluated approaches, the “Fast-precise” mode demonstrated the lowest RSS (Table S4). Moreover, it was the only method for which the transformed 1KGP distribution was statistically indistinguishable from the original RPC distribution generated using the masked T2T-CHM13 method (Fig. 5A and Fig. S10). Therefore, we chose to use the “fast-precise transformed” DJ counts for further analyses (Fig. 5A). We obtained a similar distribution as found in the RPC or UK Biobank samples, albeit with a slightly higher fraction of individuals binned in 8, 9 or 11+ DJ copies (Fig. 5A, Table S6 and Fig. S11). Here, we also observed a pattern in which the parents have a distribution shifted slightly higher than the offspring, consistent with our findings from the RPC cohort and UK Biobank samples (Fig. 5A and 5B).

Seven individuals in the 1KGP dataset were classified as having 8 DJs, suggesting possible ROB carriers (Table S7). All seven samples were identified within pedigreed families, allowing us to confirm the inheritance pattern. Among the four families, one family showed a potential ROB was inherited in the father-son relationship (HG01204-HG01206), and in the mother-daughter relationship (HG00651-HG00652). Two potential ROB carriers were found only in the parental genomes and not observed in their offspring. Interestingly, one offspring had 8.1–8.2 copies, while neither parent carried 8 DJ copies, indicative of a possible *de novo* ROB. Alternatively, given the parents are both classified as 9 copies, it is also possible that this child inherited two DJ-less acrocentric chromosomes. Unfortunately, these samples were not selected by the HPRC and so never underwent karyotyping. However, one sample, HG01204, was initially selected for sequencing by the HPRC and underwent G-banding for quality control. This revealed a karyotypic aberration involving a fusion between Chrs. 13 and 21 (Fig. S12), which is an extremely rare type of a ROB chromosomal fusion with only a few case reports available to date (for example, Chen *et al.*^31^). HPRC ultimately excluded this sample due to its abnormal karyotype, but this example highlights the ability of our method to correctly identify a true ROB from a large biobank.

Since no 8 DJ cases were sequenced by the HPRC, we evaluated all remaining cases of abnormal DJ counts. Six samples with 9 predicted DJs and ten samples with 11 predicted DJs were included in the HPRCr2 assemblies, allowing us to further inspect the sequence-level structure of the DJs and the surrounding region. To confirm the entirety of the DJ structure, we divided the DJ target into 3 regions—two palindromic arms residing close to the CER repeat and the region flanking to the rDNA (DJarms and DJflank in Fig. 5C). We searched for the DJ, rDNAs and other satellite repeats commonly found on the short arms of the acrocentric chromosomes (Fig. 5D and Table S8). Next, we investigated the assembly graph to confirm that the distal sequences were unambiguously assembled and connected to the rDNA nodes (Table S9). HiFi or ONT reads were mapped to the junction to validate the loss of DJs.

Among the 6 samples with 9 DJ copies, 3 samples successfully assembled the junction with the loss of DJ (Fig. 5D and Fig. S13). In the first case, HG00621, the DJ was lost entirely, including the HSat3 (A4) distal to the DJ and the beginning of the rDNA array. The rDNA array started at the 28S subunit rather than the regular 5’ external transcribed spacer. FISH painting of the DJs for this family showed the loss of DJ was also observed in the paternal genome, HG00619 (Fig. 5F). In the second case, HG01891, we found the DJ was also lost entirely, including part of HSat3 (Fig. 5D). The beginning of the rDNA array was found inverted with a gap at the inversion breakpoint within the array. A local re-assembly of the inverted rDNA junction was confirmed with ONT UL reads, suggesting this is a true inversion of the rDNA (Fig. S14). The chromosome painting showed the absence of DJ was also present in the maternal genome on Chr. 15 (Fig. 5F). The third case, HG01981, had DJ counts of 9.1–9.4, indicating slightly higher counts for the samples grouped as 9 DJs (Fig. 5G). We found the DJ was mostly intact except for the sequence flanking to the rDNA array on Chr. 21. Given that all 3 cases are showing non-overlapping breakpoints, we conclude that loss of 1 DJ is less common, however still transmissible within families.

When investigating the 8 samples with extra DJ junction assembled, we found 7 samples assembled the entire distal sequence to the telomere, including the duplicated DJ (Fig. 5G and Fig. S15). Within those samples, we found the second DJ was partially duplicated with the breakpoint residing internal to the flanking sequence, including distal sequences. Six samples shared a similar segmentally duplicated block spanning sequences down to the large beta satellite block. The DJ arm was truncated, with only the beginning 34 kb part being duplicated. This truncated DJ flank sequence is shared downstream the DJ, as an inverted repeat, with long-noncoding transcripts and pseudogenes. Chromosome painting of the duplicated DJ in NA20752 (GM20752) and NA20805 (GM20805) was not visible, likely due to their proximity at this scale (Fig. 5H). Arguably, one haplotype of Chr. 21 showed stronger intensity for the DJ probes, suggestive for a possible duplication. The second case, found in HG03654, had a block duplicated but starting with a smaller portion of the DJ flank down to the ACRO repeat (Fig. 5G). The last case, HG00320, was partially assembled, surrounded by rDNA sequences. In this sample, the partial DJ contained only one arm of the DJ palindromes, with the flanking sequence intact to the rDNA. Given that we see 6 cases with overlapping segmentally duplicated breakpoints ending at the same DJ flank sequence, we conclude this form is the more common form present among the extra DJ carriers.

### Computing resources and prediction model

Considering that current and past large-scale sequencing studies predominantly rely on short reads, utilizing the GRCh38 reference genome in most cases, we developed our method into a tool “DJCounter” to enable accurate detection of ROBs and determine the DJ copy numbers, all while maintaining reasonable computational demands. Here we summarize the various filtering and normalization strategies that were tested (Table S4).

Initially, we used the most conservative approach, normalizing DJ counts based on read depth from alignments to masked T2T-CHM13 (“High-resolution” mode). However, this approach proved too computationally intensive to apply to large cohorts such as the UK Biobank dataset. To address this limitation, we tested an alternative “Fast” mode that utilizes raw read counts directly from the BAM index file. While doing so, we refined the DJ target to include the DJ flank sequences that were missing in the “pilot-Fast” mode. Although computationally efficient, this method does not filter out secondary, supplementary or duplicated read alignments, resulting in inconsistent results (batch effects) depending on how the BAM file was generated. We next tested two additional modes that filter out the aforementioned reads prior to estimating DJ counts. The first option (“Fast-precise” mode) uses all filtered reads mapped to the autosomes and normalizes by the total size of the autosomes. The second option (“Fast-refine” mode) calculates the median filtered read depth across all autosomes and uses the selected autosome size for normalization.

**Figure 6 |:**
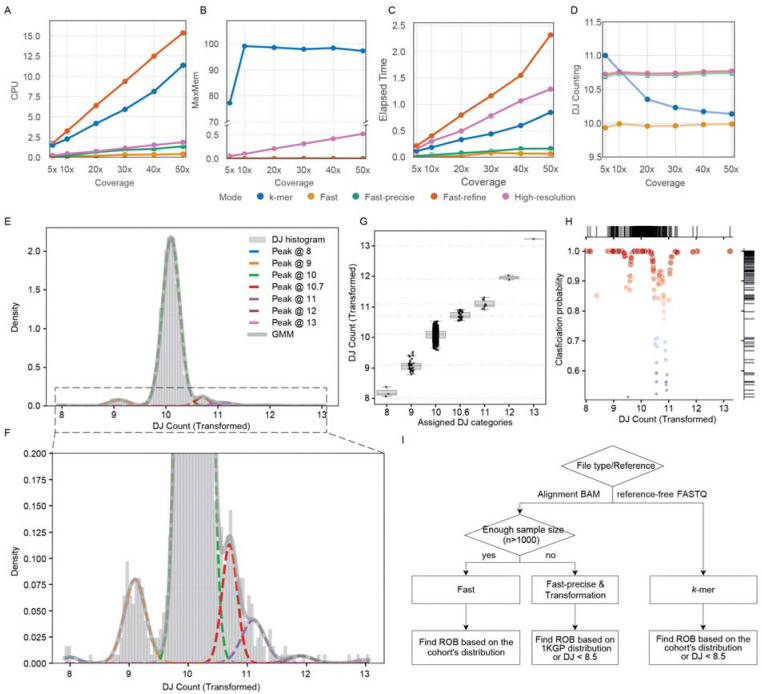
Computational resources required for DJ estimation using GRCh38 alignment–based and *k*-mer approaches, and DJ classification using a GMM trained on 1KGP distribution. (A-C) Computational resources required for DJ calculation across methods, including total CPU time in hour, maximum memory usage (MaxMem, GB) and Elapsed time (wall-clock hours). (D) DJ copy number estimation using different approaches, methods, and sequencing coverages. HG002 short read data were used and subsampled to varying depths.(E) Gaussian mixture model (GMM) fitting on the training set subsampled from 1KGP (n = 2,402). (F) Zoomed-in view of the GMM fit. (G) DJ classification using the trained GMM on the independent test subset of 1KGP (n = 800). (H) Transformed DJ copy numbers with corresponding classification probabilities for each DJ category. (I) Recommendations for using DJCounter based on input format and sample size.

We benchmarked the computational requirements of the three modes for GRCh38 and the *k*-mer based method across different sequencing depths. We subsampled the Illumina NovaSeq reads for HG002^32^ to 5×, 10×, 20×, 30×, 40×, and 50×. As expected, the “fast” mode was the most computationally efficient option, requiring substantially less CPU time and maximum peak memory (Fig. 6A-C and S16). The “fast-precise” mode utilized CPU time close to the “fast” mode, making it suitable for the next best option. The *k*-mer based method was faster than the “fast-refine” mode, utilizing the given 120 GB of memory. The *k*-mer based method operates on Meryl^33^, which can trade shorter CPU wall time for higher memory. The “fast-precise” and “fast-refine” methods show higher DJ count estimates (+0.7), however provide robust results across different sequencing coverage over 5× (Fig 6D). The *k*-mer based method reports closer to the expected 10 copies over 10×. Nevertheless, these results indicate that both mapping and *k*-mer based methods are applicable in clinical settings even for shallow sequencing data generated.

A limitation of this approach is that it requires a large cohort to accurately estimate the distribution and identify ROB samples based on that distribution. Therefore, we developed a framework that operates at a single-sample level, enabling calculation of DJ copy number directly from GRCh38 alignments and identifies ROB candidates using a reference density derived from a large cohort. We constructed a Gaussian mixture model (GMM) to transform DJ copy number estimates using subsamples from the 1KGP cohort (training set, n = 2,402) and validated the model on the remaining samples (test set, n = 800). This framework identifies candidate ROB samples and classifies each sample into a discrete copy number bin, even when only a single sample is available. Due to the uneven density across peaks, we fixed the mean of each component and constrained peak positions to align between the RPC and 1KGP cohorts (Fig S17). An additional intermediate component between 10 and 11 copies was included to reflect the partially duplicated DJ we observed (Fig. 5F) as a separate category (Fig. 6E-F). Using this model, we could assign a discrete integer bin for each sample in both the 1KGP test set (Fig. 6G) and the RPC cohort (Fig. S18), along with posterior classification probabilities. The classification probabilities indicated high confidence (>0.5) for all samples tested, with the majority of the test set showing strong support (>0.9), including ROB samples (Fig. 6H).

For optimal performance, we recommend using DJCounter under different scenarios (Fig. 6I). For studies with large sample sizes (n > 1,000), where the cohort-specific distribution can be reliably estimated and reads are aligned to GRCh38, we recommend running the “fast” mode and inspecting the overall distribution to identify potential ROB carriers. However, applying a strict cutoff of 8 copies may be challenging, as the fast mode includes duplicates and supplementary reads, which can shift the overall distribution. In contrast, for smaller cohorts (n < 1,000), where the distribution cannot be robustly inferred, the “fast-precise” mode is preferred. In this setting, the classification approach should be applied to DJ estimates generated using the fast-precise mode, with duplicate reads properly removed or marked in the alignment files. The *k*-mer based method is reference-free and does not require the alignment step. Therefore, it is recommended when rapid diagnosis is needed without alignments.

## DISCUSSION

Here we used recently completed T2T and near-T2T assemblies to understand the structure and conservation of the short arms of human acrocentrics chromosomes, particularly surrounding the rDNA arrays. Using this information, we developed a new method for genotyping the rDNA distal junction and other features of the short arms to predict the presence of Robertsonian chromosomes. Importantly, our method can be successfully applied to short-read sequencing datasets, enabling its application to large-scale biobanks, as demonstrated here using the RPC, UK Biobank, and 1KGP datasets.

Given the concordance between the observed frequency and the previously reported ROB prevalence, as well as the observed inheritance patterns, we propose that a DJ copy number of 8 or less is a reliable marker for screening ROB carriers. We also looked into the frequency distribution of the PHR on the proximal short arms of Chrs. 13, 14, and 21, where one copy is expected to be lost after ROB formation. However, a wider range of PHR copy numbers was observed, indicating the polymorphic nature of this sequence, and PHR copy number was not significantly associated with DJ copy number. Thus, in contrast to the DJ, the copy number of the PHRs does not appear to be a reliable marker of ROBs. Prior work points to the SST1 macrosatellite and an enrichment of PRDM9 motifs within the ROB-associated PHR as possible recombination hotspots, both explaining the higher prevalence of 13;14 and 14;21 ROBs, as well as the high degree of structural variation^14^. Cataloging structural variants and their implications across the acrocentric short arms is an area of future work.

The DJ and the structure of the sequences distal to the DJ on the acrocentric short arms are nearly impossible to genotype without near complete genome assemblies built from long reads. The near-T2T HPRCr2 assemblies allowed us to understand the loss and gain of the DJs, revealing previously uncharacterized structural variation in this region. The 9 DJ cases found in the 1KGP set were sporadic, with the DJ-less junction sequence not found in the RPC cohort. The gain of DJs was more frequently observed, with blocks of neighboring sequences duplicated alongside. The recurrent duplication found in 6 out of 8 cases across the 1KGP populations indicate that this is the most common form of duplication, regardless of ancestral background.

Originally, we expected the copy number of the DJs to be found as 10 in most samples, with only a handful of 8 DJs, as an indication for ROBs. However, our genotyping results from the two large cohorts showed a greater variability in the copy number, especially in the 9 or 11 range. Using the learned frequency of the 9 DJs we observed, we estimate a rough false positive rate for having two DJ-less haplotypes could be up to one third of the 8 DJ cases (Methods). Alternatively, it is also possible to miss a true ROB carrier when the individual carries the duplicated DJ in one of the non-ROB chromosomes, leading to a rough false negative rate of 7.2%. Copy number analysis alone is limited to distinguish a true ROB carrier from these alternative scenarios. Therefore, we suggest using our tool as a screening method to identify potential ROB carriers, followed by karyotype confirmation. This approach will be particularly useful for screening large cohorts to prioritize samples for confirmatory cytogenetic testing.

Our method for DJ counting and ROB prediction could have immediate clinical application, where whole-genome sequencing is commonly performed during routine carrier screening, but where Robertsonian chromosomes are not currently diagnosed^34–36^. A positive result returned by a sequencing-based method could be followed by a karyotype for confirmation, providing more sensitive screening. Furthermore, ROB carriers are typically phenotypically normal at the time of birth and most carriers go undiagnosed throughout their lives; however, aside from the fertility complications, little is known about any additional health implications. Also uncertain are the implications of DJ gain or loss, which have not been characterized prior to this study. The DJ includes important regulatory elements that have been shown with nucleolar function^37^. Thus, disruption of the DJ through either duplication or deletion may affect the transcription and function of the adjacent rDNA array. Future studies, aided by the methods and results presented here, are now needed to understand the clinical implications of structural variation on the short arms of the acrocentric chromosomes.

### Limitations

To validate the four 8 DJ individuals we identified in the RPC cohort are ROB carriers, we attempted to contact them to request participation. Despite our efforts, all individuals were unable to be recontacted or declined to participate. Additionally, due to the extensive review processes required to re-contact ROB candidates within the UK Biobank, we instead genotyped the DJ copy numbers in the widely studied 1KGP lymphoblastoid cell lines. The structural variation in the DJ region may contain variants specific to the cell line as reported in the 1KGP dataset^23^.

## RESOURCE AVAILABILITY

### Lead contact

Further information and requests for resources should be directed to and will be fulfilled by the lead contact, Arang Rhie rhiea@nih.gov and Adam M. Phillippy adam.phillippy@nih.gov.

### Materials availability

This study did not generate new unique reagents or sequencing data.

### Data and code availability

DJCounter is available on GitHub (https://github.com/marbl/DJCounter). The initial DJ counting method for the masked T2T-CHM13 version is available on GitHub (https://github.com/arangrhie/T2T-Ref). The acrocentric haplotypes presented in this study from the Verkko assemblies are available on Zenodo (https://doi.org/10.5281/zenodo.18895340) along with the acrocentric satellite annotation.

## Methods

### METHOD DETAILS

#### Samples and data used

##### Control data

***For CHM13***, PCR-free Illumina Hi-Seq 2500 paired-end reads from Nurk *et al*.^1^ were used. The reads are available on SRA: SRX1009644.

***For HG002***, PCR-free Illumina NovaSeq paired-end reads from Baid *et al*.^2^ were used. The reads are available under google storage:

https://console.cloud.google.com/storage/browser/brain-genomics-public/research/sequencing/fastq/novaseq/wgs_pcr_free/50x.

***For the three previously identified ROB samples***, PCR-free Illumina NovaSeq X paired-end reads from de Lima *et al*.^3^ were used. The reads are available under dbGap: phs003920.v1.p1.

##### Tested cohorts

###### Reverse Phenotyping Core cohort

The original Reverse Phenotyping Core (RPC) cohort data included in the present analysis consisted of BAM files per sample, originally collected as part of previously published newborn trio genomic sequencing study^4^ aligned to the hg19 reference genome. We therefore extracted all paired-end reads from the BAM files using samtools fastq and realigned them to either CHM13v2.0 or GRCh38.

samtools sort -@ $cpu -n -o ${name}_nameSort.bam ${inBam}

samtools fastq -@ $cpu ${name}_nameSort.bam \

−1 $tmp/${name}_1.fq \

−2 $tmp/${name}_2.fq

###### UK Biobank

To obtain DJ and PHR count estimates on the UK Biobank, GRCh38 alignment files in cram format stored in subfolders of the /Bulk/GATK and GraphTyper WGS/Whole genome GATK CRAM files and indices [500k release]/ path were accessed through the UK Biobank Research Analysis Platform (UKB RAP, https://www.ukbiobank.ac.uk/use-our-data/research-analysis-platform). The Fast copy number estimation method described below was then executed via the UKB RAP application called swiss-army-knife (SAK) version 4.5.0, with local and remote scripts communicating through the dxpy python library.

###### 1KGP

The 1000 Genomes Project dataset 30x Illumina NovaSeq 6000 paired-end reads were obtained from Byrska-Bishop *et al*.^5^. BAM files aligned to GRCh38 were downloaded and processed locally following instructions on IGSR for the 2504 unrelated and the 698 samples: https://www.internationalgenome.org/data-portal/data-collection/1000genomes_30x.

#### Target regions for DJ, PHR and rDNAs

Here we describe 3 features for copy number estimates of the distal junction (DJ), pseudo-homolog region (PHR) and the ribosomal DNA (rDNA).

##### Target regions in T2T-CHM13 and masked T2T-CHM13 reference

We used CHM13v2.0 as the reference genome, with the revised Cambridge Reference Sequence (rCRS) included for mitochondrial sequences. Centromeric Satellite (CenSat) repeat annotations from Altemose *et al*.^6^ were used to define target regions of interest. For identifying internal repeat structure, we used ModDotPlot v0.9.0^7^ to visualize sequence identity and its orientation.

***The DJ region*** spans ~341 kbp upstream of the rDNA arrays in the short arms of the acrocentric chromosomes. We defined the DJ excluding the CER repeat as it was identified in the CenSat annotation from Nurk *et al*.^1^ and Altemose *et al*. 2023^6^. After comparing the DJs on T2T-CHM13v2.0 to each other, we found minor variations on each unit, however the majority stayed concordant (Fig. S1-2). The DJs were all similar in terms of length (341,898–346,124 bps) with high identity (>95%), except for the bSat between the DJ arm and DJ flank (Fig. 5C) and the variation found within the second DJ arm as reported before^8^.

***For the PHR***, we used the region as presented in Guarracino *et al*.^9^ and refined the boundaries to exclude the inverted repeat. The PHR on Chr. 13 is the longest, spanning 1,174,544 bps, while Chr. 14 and Chr. 21 have a shorter form, spanning 1,124,403 and 1,126,601 bps, respectively. We chose the longest form on Chr. 13 as the target for the PHR. The PHR consists of two regions outside the highly repetitive satellite repeats, which we refer to here as PHR arm1 and PHR arm2 (Fig. S3).

***For the rDNA***, we compared the first rDNA units in each chromosomal array (Fig. S4).

Among them, the unit on Chr. 13 had the longest form and was therefore chosen.

The final target regions for the DJ, PHR arm1 and the first rDNA unit are on Chr. 13. We masked the rest of the units present elsewhere in the reference using bedtools maskfasta (v 2.31.1). Although not used in this study, we additionally masked all 5S rDNA units except the last copy on Chr. 1.

To account for differences in sex chromosome complement, we applied the sex chromosome complement reference (SCC) approach to ensure proper read mapping on the sex chromosomes. Specifically, we used a pseudoautosomal region (PAR)-masked Y reference for XY individuals and a Y-masked reference for XX individuals. Table S1 contains the exact coordinates of the target and masked region used on T2T-CHM13v2.0.

##### Target regions on GRCh38

To establish target coordinates on GRCh38, we extracted the corresponding target sequences from CHM13 and aligned the GRCh38 reference genome (https://rcs.bu.edu/examples/bioinformatics/gatk/ref/) to each target sequence using Minimap2 (v2.30)^10^ with the -ax asm20 option. The resulting alignments were converted to PAF format using paftools.js (sam2paf). Regions shorter than 10 kbp were excluded from further analysis (Tables S2-4).

minimap2 -ax asm20 {target}.fasta Homo_sapiens_assembly38.fasta.gz

The PHR regions were defined as described above, resulting in a single location found on chr21:9722154–9796322 (+). Additional inspection was performed to refine the DJ region in GRCh38.

Initially, we evaluated the palindromic region of the DJ (ct_13_9) for counting the DJs. Before applying this approach to the larger cohort, we selected 50 samples from RPC and aligned the reads to GRCh38. Paired-end short reads were aligned to the GRCh38 reference using the same pipeline that we applied to RPC samples aligned to CHM13 (see “Read alignment on each reference” below). Next, we inspected the base-level sequencing depth across the DJ region by lifting over the alignments from GRCh38 to CHM13. This was done using the target and query coordinates in the PAF file, scaling the query’s start and end positions to match the full DJ coordinates.

Per–base-pair depth was calculated using samtools depth. Following manual inspection, a subregion within the original DJ target in CHM13 (chr13:5,566,000–5,600,000) was excluded due to copy number variable alignment behavior in GRCh38. The refined DJ target coordinates were subsequently lifted back to GRCh38 to generate a final BED file for copy number estimation (Table S2). We found that using this target produced the expected distribution compared to the samples distribution on T2T-CHM13, with the majority of samples exhibiting approximately 10 DJ copies. This BED file was used to assess DJ copy number variation across UK Biobank samples.

However, we later found that extending the DJ target region to include upstream and downstream of the initial ct_13_9 sequences encompassing the full DJ and its flanking regions resulted in a stronger correlation with the CHM13-based mapping results. Regions on chromosomes 4 and 10 with low sequence identity (<83.4%) and low coverage (<2.8%) were excluded from analysis. Therefore, this expanded region was selected as the final region of interest for DJ copy number estimation.

#### Read alignment on each reference

##### CHM13

###### Pilot testing on the control samples

We used Illumina short reads from the 3 identified ROB samples from de Lima *et al*^3^, and mapped them to the masked reference. As a control, we used Illumina short reads from CHM13 and HG002, which were previously identified to have 10 DJs from FiSH experimental data (Nurk *et al* and Supplementary Figure XVIII.59 in Yoo *et al*.)^1,11^.

###### RPC

We utilized the read coverage on chromosome X and Y in the original GRCh37 alignments to infer the sample’s biological sex. First, we confirmed the BAM file is sorted based on genomic coordinates. Next, we used samtools idxstats to calculate the number of mapped reads to each sex chromosome and collected the size of the sex chromosomes. The coverage for chromosomes X and Y was determined by dividing the total number of mapped reads by the total chromosome length for each respective chromosome. To infer the sample’s sex, we calculated the coverage ratio between chromosome X and chromosome Y (covX/covY).

Samples with a ratio greater than 4 were classified as XX, while those with a ratio of 4 or less were classified as XY. Once the presence of Y was confirmed, the proper masked reference has been set to be used in the downstream analysis.

Short-read paired-end data from both the control samples and the RPC were aligned to the corresponding SCC reference using BWA (v0.7.17)^12^ and post-processed with samtools (v1.21)^13^ using fixmate, sort and markdup. Secondary alignments were removed with samtools view -F0×100 option.

bwa mem -t $cpu $ref $r1 $r2

samtools fixmate -m -@$cpu $out.sam $out.fix.bam

samtools sort -@$cpu -O bam -o $out.bam -T $out.tmp $tmp/$out.fix.bam

samtools markdup -r -@$cpu $out.bam $out.dedup.bam

#### GRCh38

To match with the method used in the UKbiobank and 1KGP, the paired-end reads from the six control samples and the 92 subsampled RPC samples were aligned to the reference genome using BWA-MEM (v0.7.17)^12^. The resulting SAM files were processed with SAMBLASTER (v0.1.26)^14^ to add mate tags and sort reads by query name, after which duplicate reads were marked using Picard *MarkDuplicates* (v3.3.0).

#### Copy number estimate

##### Mapping based approaches

###### T2T-CHM13v2.0

Copy number estimates for target regions were calculated as the ratio of median sequencing depth in the target region to that of the autosomal background, excluding acrocentric short arms. Median depth for each target and background region was computed using samtools (v1.23)^13^ and bedtools (v2.31.1)^15^ with the following command:

samtools depth -@ $cpu -b $bed $bam | \

 awk ‘{print $1,$2–1,$2,$3}’ OFS=‘\t’ - | \

 bedtools map -a $bed -b - -c 4 -o median > $prefix.coverage_results.bed

For a given target T, the copy number $\left(CN_{T}\right)$ was calculated as:

$$CN_{T}=2\times \frac{Median\left(Depth_{T}\right)}{Median\left(Depth_{B}\right)}$$

where $Depth_{T}$ represents the depth per base-pair within the target region and $Depth_{B}$ represents the depth across the autosomal region.

###### GRCh38

Challenges in the normalization process arose from collapsed, misassembled, and fragmented target sequences in the acrocentric short arms, in particular the DJ regions in GRCh38, as well as from potential PCR-duplicate–like biases observed in reads mapped to these regions. To reduce computational bottlenecks and minimize the impact on the target copy number estimation, we evaluated four different approaches for calculating the target and background sequencing depth.

Starting from the pilot-Fast mode, we developed four approaches and benchmarked the 92 RPC samples and compared correlations of the distribution to the result from T2T-CHM13 (Fig. S7).

***For the pilot Fast mode applied to the UK Biobank cohort,*** we used samtools (v1.20)^13^ to collect the number of reads mapped to the target and all regions. Specifically, we used samtools view -c aligned to the target (*RC*_*T*_, Table S2 for the DJ target) and samtools idxstats to collect reads aligned to the autosomes by using num. of mapped reads − num. of unmapped reads. In this mode, no reads are filtered prior to calculation. Unlike the RPC samples, we learned samtools depth was creating a bottleneck and was too costly to run on all 490k samples on DNAnexus (1 hour vs. 2 minutes using samtools idxstats). Albeit a little shifted, we found a good correlation to the T2T-CHM13 based result for the 92 subsamples (Pearson’s correlation, r=9.81, p=57×10^−66^) between the two methods (Fig. S8), and decided to move forward using this approach. The script was deployed on DNAnexus and collected copy counts of the DJ, rDNA, and PHR.

Copy number for each target was estimated using read count normalization. Read counts (RC) mapped to each target were normalized to autosomal coverage, which was calculated by subtracting unmapped read counts $\left(RU_{A}\right)$ from the total reads mapped to autosomes $\left(RC_{A}\right)$ and dividing by the total autosomal length $\left(Len_{A}\right)$. The normalized target RC was then used to infer copy number.


$$CN_{GRCh38-pilotFast}=2\times \frac{SUM\left(RC_{T}\right)/Len_{T}}{SUM\left(RC_{A}-RU_{A}\right)/Len_{A}}$$

After a series of evaluations, we updated our method to include the DJ flanking region in the target as it yielded better copy number estimates. The following four modes (Table S5) use the target DJ region as shown in Table S3.

***The Fast mode*** estimates copy number using the number of reads aligned to the target region (*RC*_*T*_) calculated by samtools view -c, normalized by the original target length in CHM13v2.0 $\left(Len_{T}\right)$. Autosomal coverage is calculated by using the median of autosomal read coverage, defined as the total number of reads mapped to each autosome $\text{(}RC_{C}\text{)}$ divided by the length of the autosomal $\left(Len_{C}\right)$ reported by using samtools idxstat. In this mode, no reads are filtered prior to calculation.


$$CN_{GRCh38-Fast}=2\times \frac{SUM\left(RC_{T}\right)/Len_{T}}{Median\left(RC_{C}/Len_{C}\right)}$$

***In the Fast-precise mode,*** reads marked as duplicates, as well as secondary and supplementary alignments, were excluded from analysis. Target coverage was calculated as the total filtered read counts aligned to the target region $\left(FRC_{T}\right)$ (as reported by samtools view -c -F 3332) divided by the target length $\left(Len_{T}\right)$. Background coverage was calculated as the total number of filtered reads aligned to the autosomes $\left(FRC_{A}\right)$ divided by the total autosomal length $\left(Len_{A}\right)$.


$$CN_{GRCh38-Fast-precise}=2\times \frac{SUM\left(FRC_{T}\right)/Len_{T}}{FRC_{A}/Len_{A}}$$

***The Fast-refine mode*** uses the same approach to calculate target coverage. However, background coverage is defined as the median coverage across autosomal regions rather than coverage calculated from the entire autosome.


$$CN_{GRCh38-Fast-refine}=2\times \frac{SUM\left(FRC_{T}\right)/Len_{T}}{Median\left(FRC_{C}/Len_{C}\right)}$$

***The High-resolution mode*** calculates coverage at per-base resolution, consistent with the approach used for CHM13v2.0. The depth for each target region was computed using samtools depth $\left(Depth_{T}\right)$ and normalized by the target length $\left(Len_{T}\right)$. For background coverage, we used samtools coverage to obtain coverage metrics for each autosome $\left(Cov_{C}\right)$ and defined the background as the median autosomal coverage.


$$CN_{GRCh38-High-resolution}=2\times \frac{SUM\left(Depth_{T}\right)/Len_{T}}{Median\left(Cov_{C}\right)}$$

#### Reference-free k-mer based approach

***For identifying the DJ k-mers,*** 31-mers were collected with Meryl (v1.4.1)^16^ for each of the five DJ copies in T2T-CHM13v2.0. The DJ sequence was extracted with samtools faidx, and meryl count k=31 was used to build the dj.$chr.meryl for each chromosome. Only *k-*mers present once in each chromosome were selected and intersected, to ensure we use *k-*mers present once in each DJ and in all 5 DJs. This yielded 178,731 potential target *k*-mers in dj.meryl.

meryl intersect [ equal-to 1 dj.chr13.meryl ] [ equal-to 1 dj.chr14.meryl ] output dj.chr13_chr14.1.meryl

meryl intersect [ equal-to 1 dj.chr15.meryl ] [ equal-to 1 dj.chr21.meryl ] output dj.chr15_chr21.1.meryl

meryl intersect [ intersect dj.chr13_chr14.1.meryl dj.chr15_chr21.1.meryl ]

[ equal-to 1 dj.chr22.meryl ] output dj.meryl

***For identifying the DJ target k-mers,*** the DJ *k*-mers were filtered one more time after projecting the k-mer multiplicity as found in the short reads. The 31-mer meryl DB was built with meryl count k=31, and the histogram was generated with meryl histogram. The 2 copy peak was inferred using GenomeScope 2.0^17^ with the following command line:

Rscript $tools/genomescope2.0/genomescope.R -i chm13.k31.hist -o chm13.k31.genomescope2 --fitted_hist --testing -k 31

The predicted haploid coverage (kcov) resulted in 49.9. The inspected 10 copy peak in CHM13 is therefore ~50 × 10; resulting in ~500x. This matched the peak distribution of the DJ *k*-mers when projected to the read spectrum (Fig. 1E). The final selected DJ target *k*-mers were chosen falling between the range of 475–525x (±0.5 copy), to allow room for small variations or sequencing biases. This resulted in 52,227 distinct DJ target *k*-mers in dj.target.meryl.

meryl greater-than 475 [ less-than 525 dj.chm13_count.meryl ]\ output dj.target.meryl

***For copy number estimate the DJs in a given sample,*** we first collect 31-mers from the samples’ short reads. The 31-mer database is then queried to collect the k-mer multiplicity histogram of the DJ target *k*-mers. The median of the DJ target k-mers found in the reads is used as the *k*-mer frequency of the DJs, and the “2-copy peak” is inferred from the histogram of all 31-mers in the reads. The 2-copy peak is obtained using a script in Merqury (kmerHistToPloidyDepth.jar), which differentiates the slope of the *k*-mer histogram and reports the estimated ploidy and its peak and boundary by collecting the local maxima^16^.

# Collect 31-mers from reads
meryl count k=31 output $sample.k31.meryl $sample.fq.gz
# Intersect DJ target k-mers
meryl intersect threads=24 $sample.k31.meryl dj.target.meryl output
$sample.k31.DJ.meryl
# Histogram of the DJ kmers found in $sample
meryl histogram $sample.k31.DJ.meryl > ${sample}_DJ.hist
# Median
count=`cat ${sample}_DJ.hist | awk ‘{if (NR>1) { count+=$NF;} } END {print count/2}’`
med=`cat ${sample}_DJ.hist | \
 awk -v cnt=$count ‘{cnt_sum+=$NF; if (NR>1 && cnt_sum > cnt) {print $(NF-1); exit;} }’`
# 2-copy peak
java -jar -Xmx256m $MERQURY/eval/kmerHistToPloidyDepth.jar $sample.k31.hist
| tail -n1 | awk ‘{print $2}’


$$CN_{k-mer}=2\times \frac{Median\text{(}DJ\;target\;k-mer\;counts\text{)}}{2-copy\;peak}$$

#### Chromosome spreads and Fluorescent In-Situ Hybridization (FISH) for rDNA and DJ

Human lymphoblastoid cell lines (LCL) and fibroblast cell lines were according to Coriell protocols. CHM13 immortalized using human telomerase reverse transcriptase (hTERT) were propagated in DMEM:F12 with L-glutamine supplemented with 1x non-essential amino acids (Gibco), 1x Insulin-Transferrin-Selenium (Gibco), 1mM sodium pyruvate (Gibco), and 10% FBS. For preparation of chromosome spreads, cultures were blocked in mitosis by the addition of Karyomax colcemid solution (0.1 μg/ml, Thermo Fisher) for 6–7h. Adherent fibroblast cells were collected by trypsinization. Cells were incubated in hypotonic 0.4% KCl solution for 12 min and prefixed by addition of methanol:acetic acid (3:1) fixative solution (1% total volume). Pre-fixed cells were spun down and then fixed in Methanol:Acetic acid (3:1). Chromosome spreads were dropped on a glass slide and incubated at 65°C overnight. Before hybridization, slides were treated with 0.1mg/ml RNAse A (Qiagen) in 2xSSC for 45 minutes at 37°C and dehydrated in a 70%, 80%, and 100% ethanol series for 2 minutes each. Slides were denatured in 70% deionized formamide/2X SSC solution pre-heated to 72°C for 1.2–1.5 min. Denaturation was stopped by immersing slides in 70%, 80%, and 100% ethanol series chilled to −20°C. Probes used in combinations were denatured in a hybridization buffer (Empire genomics) by heating to 80°C for 7 minutes before applying to denatured slides. Specimens were hybridized to the probes under a glass coverslip or HybriSlip hybridization cover (GRACE Biolabs) sealed with the rubber cement or Cytobond (SciGene) in a humidified chamber at 37°C for 48–72hours. After hybridization, slides were washed in 50% formamide/2X SSC 3 times for 5 minutes per wash at 45°C, then in 1x SSC solution at 45°C for 5 minutes twice and at room temperature once. For biotin detection, slides were incubated with streptavidin conjugated to Cy5 (Thermo) for 2–3 hours in PBS containing 0.1% Triton X-100 and 5% bovine serum albumin (BSA), and then washed 3 times for 5 minutes with PBS/0.1% Triton X-100. Slides were rinsed in water, air-dried in the dark, and mounted in Vectashield containing DAPI (Vector Laboratories). Z-stack confocal images were acquired on the Nikon TiE microscope equipped with 100x objective NA 1.45, Yokogawa CSU-W1 spinning disk, and Flash 4.0 sCMOS camera (Hamamatsu). Image processing was performed in FIJI.

#### Pattern of DJs in the HPRC samples

##### HPRC Verkko assemblies

In parallel to the HPRCr2 assemblies, a Verkko v2.2.1^18^ assembly of each genome was made using automated scripts to download data from AWS, build parental k-mer databases, and run Verkko with both Hi-C and Trio data in parallel. Standardized post-processing and QC was performed with the same pipeline which used Yak (https://github.com/lh3/yak) to measure QV (k=31) and switch error rate, compleasm^19^ for gene completeness, and computed T2T contig and scaffold statistics using seqtk to identify the telomeric and gap sequences in the assemblies. For each sample where a trio and Hi-C was available, the assembly with the most T2T contigs was selected. In total, there were 59 trios and 159 Hi-C assemblies.

##### Chromosome assignment and orientation

Mashmap v3.1.1^20^ was used to map the assembly to CHM13v2.0. The contigs were assigned to chromosomes if they had mappings over 99% identity covering more than 10% of the reference chromosome in a given orientation (forward or reverse-complement). If a contig matched over 10% of more than one chromosome, multiple assignments were noted and the longest one was used. The XY pseudo-autosomal regions (PAR) and distal regions of the acrocentric were excluded from the covered fraction count as they recombine between individuals and cannot be accurately assigned via reference alignment. Therefore, these regions could only be named as a chromosome if they were assembled together with a unique region of the chromosome. Across all samples and assembly types (verkko-hi-c or verkko-trio+hi-c), there were 23,094 contigs totaling 2.11 Tbp with an average size of 91.58 Mbp which could be oriented and assigned. There were 338 contigs with ambiguous assignments with an average size of 5.7 Mbp and a total of 1.936 Gbp while 75,565 contigs could not be assigned with an average size of 0.12 Mbp totaling 8.73 Gbp.

In parallel, the homopolymer-compressed Verkko gfa were aligned using Mashmap (v3.1.1)^20^ to a compressed version of CHM13v2.0 to assign the individual nodes to a chromosome. A two pass assignment was performed. First, nodes with mappings over 99% identity and 5 Mbp matches to a chromosome were assigned to that chromosome. Second, if a connected component had no nodes assigned by the first criteria, nodes over 500 kbp were assigned. This identified connected components in the assembly graph belonging to each chromosome.

##### Repeat annotation of the acrocentric chromosomes

The acrocentric chromosomal short arms carry human satellite repeats (HSat) at and around the centromere, some specific to the acrocentric region forming a particular pattern^1,21^. To obtain an overview of the sequence content and confirm sequences belonging to the acrocentric short arms, particularly the distal and proximal sequences from the rDNA, satellite sequences in the assemblies were annotated based on a set of targeted sequence (Table S8).

In brief, the target repeat sequences were selected based on the CenSatv2.1 annotation available for T2T-CHM13v2.0 (https://s3-us-west-2.amazonaws.com/human-pangenomics/T2T/CHM13/assemblies/annotation/chm13v2.0_censat_v2.1.bed). Most were selected from chr13 or chrY, except for HSat1A, HSat3_A5, and HSat3_B3. For these three HSat repeats, the original consensus generated for classifying the satellite array subgroups were used as described by Altemose *et al*.^22^. The consensus sequences are available as fasta files on https://github.com/altemose/HSatReview/tree/main. The rDNA reference KY962518 was rotated to begin upstream of the 45S TSS, to include the 45S promoter region.

The target sequences were found using minimap2 (v2.28) in each assembly. Because most mappers are designed to find one best match for each query in the reference, using the assembly as the reference would yield only one result for each mapped target satellite. Thus, the reference and query sequences were flipped, mapping the assembly (query) to the targeted repeat sequence (reference) instead. This approach allows a more sensitive search of the target sequence in the assembly. Once all the alignments are collected in a PAF file, the reference and query coordinates were inverted with rustybam (v0.1.33)^23^ and converted to BED format. Alignment blocks within 500 bp were merged with BEDTools (v2.31.1)^15^ and formatted with designated colors and filtered to be over 2 kbp or 7–8 kbp to exclude excessive alignment matches to LINE elements.

Below are the command lines used:

minimap2 -x asm20 --eqx --MD -t $cpus -c $target.fa $asm_fa >>
${sample}_to_sat.paf
rustybam invert ${sample}_to_sat.paf > sat_to_${sample}.paf
cat sat_to_${sample}.paf |\
 awk -v OFS=‘\t’ ‘{if ($10<$11) {idy=100*$10/$11;} \
  else {idy=100*$11/$10;} print $6,$8,$9,$1,idy,$5}’ |\
 sort -k1,1V -k2,2n - > sat_to_${sample}.bed

For each target satellite, merging and filtering was applied along with the color assignment. Telomere sequences and gaps were annotated with Seqtk (v1.4, https://github.com/lh3/seqtk) using tel -d5000 and gap -l 1 parameters.

awk -v chr_hap=$chr_hap -v sat=$sat ‘$1==chr_hap && $4==sat’
${out}/sat_to_${sample}.bed | \
 bedtools merge -s -d 500 -c 4,5,6 -o distinct,median,distinct -i - |\
 awk -v len=$len ‘$3-$2>len’ |\
 awk -v chr=$chr -v col=$col -v OFS=“\t” ‘{print chr, $2, $3, $4, $5, $6, $2,
$3, col}’ >> $OUT

The final BED file was then concatenated and sorted for manual inspection and visualization on IGV (v2.18.2)^24^.

#### Benchmarking and optimizing DJCounter for GRCh38

##### Subsampling and benchmarking computing resources

HG002 reads aligned to GRCh38 were subsampled to the desired sequencing depths (1×, 5×, 10×, 20×, 30×, 40×, and 50×) using samtools (v1.21)^13^ to evaluate the stability and computational requirements of each method. We ran DJCounter on the subsampled BAM files using the GRCh38-based modes and additionally extracted reads from the BAM files for analysis with the k-mer–based method.

To benchmark computational requirements of each DJ estimation mode, we extracted runtime and resource utilization metrics from SLURM job accounting records using sacct. For each job, we summarized: (i) Elapsed time as the maximum wall-clock duration across steps (hours); (ii) Total CPU time as the sum of CPU time across steps (hours); (iii) maxMem as the maximum resident memory usage (MaxRSS, GB); (iv) maxVmem as the maximum virtual memory usage (MaxVMSize, GB); (v) %CPU as the average CPU utilization relative to a single fully utilized core (100%); and (vi) AvgCPU as the mean CPU time per step (hours).

#### Linear transformation between GRCh38- and CHM13-derived DJ copy numbers

To determine which GRCh38 mapping mode exhibited the least cohort bias and to harmonize DJ copy number estimates derived from GRCh38 alignments with those obtained from CHM13, we fitted an ordinary least squares (OLS) linear regression model using the *statsmodels* package (v0.14.5)^25^. Specifically, DJ copy numbers calculated from CHM13 alignments of 92 RPC subsamples were modeled as a function of DJ copy numbers derived from GRCh38 alignments of the matched samples:

$$DJ_{CHM13}=\beta_{0}+\beta_{1}\times DJ_{GRCh38}+\epsilon$$

where $\beta_{0}$ represents the intercept, $\beta_{1}$ the slope, and *ϵ* the residual error term.

We fitted this model separately using DJ copy numbers generated from different GRCh38 modes (Fast, Fast-precise, Fast-refine, and High-resolution) and selected the Fast-precise mode because it yielded the lowest residual sum of squares (RSS) among all modes and showed no significant difference between the CHM13-aligned RPC DJ distribution and the transformed 1KGP DJ distribution.

To harmonize DJ copy numbers across cohorts, we applied the fitted Fast-precise regression model to transform GRCh38-derived DJ estimates to the CHM13 scale using the `predict` function in *statsmodels*. The transformed values were subsequently used for GMM fitting and classification analyses

##### GMM fitting and classification

We used the transformed DJ copy numbers to split the 1KGP samples into a training set and a test set at a ratio of 0.75 and 0.25, respectively. When applying a standard GMM without fixed means, the model failed to identify peaks in sparsely populated regions and exhibited over-dispersion or component collapse, particularly for rare categories such as ROB samples with 8 copies. To address this issue, we performed GMM fitting with fixed component means and constrained standard deviations (0.1≤σ≤0.2).

Candidate peak locations were identified from the DJ copy number distributions of the RPC cohort aligned to CHM13 and the transformed 1KGP (GRCh38-aligned) cohort using the `find_peak` function from the *scipy.signal* module (v1.13.1)^26^. Peaks that were consistent across cohorts were selected as 7 $\left(k\right)$ fixed component means $\left(u\right)$: [8, 9.1, 10.1, 10.7, 11.1, 11.9, 13].

To quantify uncertainty in the estimated mixture parameters, we performed nonparametric bootstrap resampling on the training set. Using the DJ copy numbers from the training data, we generated B=1,000 bootstrap datasets by sampling observations with replacement. For each bootstrap replicate b, we fit a one-dimensional GMM with fixed means and constrained standard deviations using an expectation–maximization (EM) algorithm. For each replicate, we estimated the mixture weights $\text{(}\psi_{kb}\text{)}$ and standard deviations $\text{(}\sigma_{kb}\text{)}$ for each k. Model fitting was performed with a maximum of 500 iterations, and convergence was defined as a change in log-likelihood of less than 1×10e-10 times between successive iterations. Final parameter ( $\psi_{k}$. and $\sigma_{k}$) estimates were obtained by averaging the bootstrap estimates of weights and standard deviations.

For classification, we used the fitted GMM parameters to assign samples from the test set to discrete DJ copy number categories. For each sample, the unnormalized posterior density for each component was computed as. $\psi_{k}\cdot norm\text{.}pdf\left(x_{i},u_{k},\sigma_{k}\right)$ and normalized across components to obtain posterior membership probabilities. Each sample was assigned to the category with the highest posterior probability (maximum a posteriori classification).

#### False positive and false negative rate

##### False positive rate.

We assume ROB carriers have 8 DJ copies based on observations from prior studies. However, as shown in our study, we found 1 DJ-less haplotypes (9 DJs) across different cohorts at a frequency of 3.1%. Assuming the 10 acrocentric chromosomes are independent, this gives us a rough estimate that each acrocentric chromosome can lack a DJ with probability of 1 - (1 – 0.031)^1/10^ ≈ 0.31%. As such, the probability of a deletion of DJs is 0.0031, the probability that exactly 2 of them have a deletion is given by

$$P(FPROB)={\left(0.0031\right)}^{2}\times {\left(1-0.0031\right)}^{8}\times C(10,2)$$

This corresponds to approximately 0.04% of all samples. This could consist up to ⅓ of detected 0.12% 8 DJ cases.

False negative rate. Given the high frequency we observed for one or more DJ gains (~8.9%), it is also possible that a ROB carrier can be misclassified as having 9 copies when one of the non-fused acrocentric haplotype has the duplicated DJ present. Similarly as above, we can roughly estimate the probability for having a duplicated DJ in each acrocentric chromosome as 1 - (1 – 0.089)^1/10^ ≈ 0.0093. Thus, the chance that a ROB carrier will have duplicated DJ in at least one of the other acrocentric chromosomes and thus more than 8 DJ can be estimated as

$$\text{P}(\text{FN ROB}|\text{ROB})=1-{\left(1-0.0093\right)}^{8}\approx 0.07$$

As a result, we expect that roughly 7% of ROBs can be missed by using 8 DJs as the sole marker for identifying ROB carriers.

Copy number analysis alone cannot distinguish between a true ROB carrier and this alternative scenario. To determine whether samples with 8 copies carry a genuine fusion chromosome, additional analysis such as karyotyping or Hi-C are required. Given that we did not observe any offspring with both parents having 9 DJs in the UK Biobank dataset, we estimate the chances might be even lower. We believe a more dedicated study on familial participants with identified 8 DJ offspring and 9 DJ parents may answer this question.

## Supplementary Material

Supplement 1

Supplement 2

## Figures and Tables

**Table 1.** Pipeline confirms 8 DJs in 3 known ROB cell lines and two control samples, where the DJ was karyotyped before. Remarks are from Coriell. *Reported from de Lima *et al*.^14^

Sample	Sex	T2T-CHM13	GRCh38	k-mer	Remarks	Age
CHM13	XX	10.3	10.1	10.2	CHM13hTERT cell line, karyotypically normal	na
HG002	XY	10.1	10.7	10.1	GM24385 cell line, karyotypically normal	na
GM03417	XX	8.3	8.4	8.3	Clinical features of Down syndrome. Mosaic; 32% of cells are balanced 45,XX, t(14;21); *Cellular heterogeneity with regard to the number of copies of Chr. 21, with 3 copies of Chr. 21 being relatively rare (~10%)	15
GM03786	XX	8.0	8.1	8.0	Clinically normal	19
GM04890	XX	8.1	8.3	8.1	Clinically normal; has had 5 miscarriages	32

**Table 2.** ROB candidates from the RPC cohort. Estimated num. of DJs, PHRs, and rDNAs are shown below with the categorized copy counts binned. ROB candidates are highlighted with a green background.

Family	Sex	DJ	Bin	PHR	Bin	rDNA	Relationship
A	XX	8.16	8	3.87	4	386.6	Mother
	XY	10.09	10	4.85	5	433.8	Father
	XY	8.88	9	4.71	5	415.6	Child
B	XX	10.2	10	6.00	6	338.3	Mother
	XY	8.04	8	4.04	4	229.6	Father
	XX	9.10	9	5.40	5	357.7	Child-A
	XX	8.95	9	5.31	5	334.8	Child-B
C	XX	8.05	8	4.77	5	254.7	Mother
	XY	9.04	9	4.85	5	325.6	Father
	XX	8.04	8	5.80	6	316.2	Child-A
	XX	9.15	9	4.90	5	424.9	Child-B

**Table 3.** Copy number estimates of the DJs across the RPC cohort (n=4,187) and UK Biobank WGS cohort (n=490,369). Regardless of the cohorts, estimated ROBs with 8 DJs had the smallest fraction of 0.11–0.12%. The largest fraction of samples were observed with 10 DJs (87.17–88.70%), followed by 11 DJs (7.81–9.00%), 9 DJs (2.81–3.45%), and more than 11 DJs (0.26–0.56%), indicating loss of one or gains of one or more DJs are relatively common.

Cohort	Num. of DJs	Num. of samples	%	XX	%	XY	%
RPC	<8	0	0.00%	0	0.00%	0	0.00%
	**8**	**4**	**0.10%**	**3**	**0.14%**	**1**	**0.05%**
	9	140	3.34%	81	3.82%	59	2.86%
	10	3,659	87.39%	1,848	87.05%	1,811	87.74%
	11	373	8.91%	186	8.76%	187	9.06%
	>11	11	0.26%	5	0.24%	6	0.29%
	Total	4,187	100.00%	2,123	100.00%	2,064	100.00%
RPC (Parents only)	<8	0	0.00%	0	0.00%	0	0.00%
	**8**	**3**	**0.11%**	**2**	**0.15%**	**1**	**0.08%**
	9	90	3.45%	51	3.91%	39	2.98%
	10	2,277	87.17%	1,135	87.04%	1,142	87.31%
	11	235	9.00%	113	8.67%	122	9.33%
	>11	7	0.27%	3	0.23%	4	0.31%
	Total	2,612	100.00%	1,304	100.00%	1,308	100.00%
UK Biobank	<8	9	0.002%	6	0.002%	3	0.00%
	**8**	**583**	**0.12%**	**312**	**0.12%**	**271**	**0.12%**
	9	13,778	2.81%	7,460	2.80%	6,318	2.82%
	10	434,951	88.70%	235,927	88.70%	199,024	88.70%
	11	38,291	7.81%	20,791	7.82%	17,500	7.80%
	>11	2,757	0.56%	1,500	0.56%	1,257	0.56%
	Total	490,369	100.00%	265,996	100.00%	224,373	100.00%

**Table 4.** Copy number estimates of the PHRs across the RPC cohort (n=4,187) and UK Biobank WGS cohort (n=490,374).

Cohort	Num. of PHRs	Num. of samples	%	XX	%	XY	%
RPC	<4	3	0.07%	0	0.00%	3	0.15%
	4	261	6.23%	126	5.93%	135	6.54%
	5	1,645	39.29%	828	39.00%	817	39.58%
	6	2,189	52.28%	1,127	53.09%	1,062	51.45%
	7	85	2.03%	39	1.84%	46	2.23%
	>7	4	0.10%	3	0.14%	1	0.05%
	Total	4,187	100.00%	2,123	100.00%	2,064	100.00%
RPC (Parents only)	<4	1	0.04%	0	0.00%	1	0.08%
	4	143	5.47%	67	5.14%	76	5.81%
	5	931	35.64%	453	34.74%	478	36.54%
	6	1,477	56.55%	755	57.90%	722	55.20%
	7	59	2.26%	28	2.15%	31	2.37%
	>7	1	0.04%	1	0.08%	0	0.00%
	Total	2,612	1	1,304	100.00%	1,308	100.00%
UK Biobank	<4	234	0.05%	128	0.05%	106	0.05%
	4	33,964	6.93%	18,195	6.84%	15,769	7.03%
	5	185,625	37.85%	100,601	37.82%	85,024	37.89%
	6	262,723	53.58%	142,803	53.69%	119,920	53.45%
	7	7,631	1.56%	4,164	1.57%	3,467	1.55%
	>7	197	0.04%	104	0.04%	93	0.04%
	Total	490,374	100.00%	265,995	100.00%	224,379	100.00%

**Table:** Key resources table

REAGENT or RESOURCE	SOURCE	IDENTIFIER
**Oligonucleotides and Probes**		
DJ OligoMix - 5’ Biotin L	LC Sciences, LLC	Custom 100bp oligo paint corresponding to CHM13 chr13:5424523–5770548
CenSat D14Z1/D22Z1	Cytocell	LPE 014
Human whole chromosome paint (chr15)	Applied Spectral Imaging	FPRPR0167
45S rDNA probe	Empire genomics	RP11–450E20
**Experimental models: Cell lines**		
CHM13	Magee-Womens Hospital (Pittsburgh) IRB MWH-20–054	CHM13
HG002	Coriell Institute for Medical Research	GM24385
GM03417	Coriell Institute for Medical Research	GM03417
GM03786	Coriell Institute for Medical Research	GM03786
GM04890	Coriell Institute for Medical Research	GM04890
HG01204	Coriell Institute for Medical Research	HG01204
HG00619	Coriell Institute for Medical Research	HG00619
HG00620	Coriell Institute for Medical Research	HG00620
HG01981	Coriell Institute for Medical Research	HG01981
HG01890	Coriell Institute for Medical Research	HG01890
HG01889	Coriell Institute for Medical Research	HG01889
GM02752	Coriell Institute for Medical Research	GM02752
GM20805	Coriell Institute for Medical Research	GM20805
**Software and algorithms**		
Bedtools (v2.31.1)	Aaron R. Quinlan *et al.*	https://bedtools.readthedocs.io/en/latest/
BWA-MEM (v0.7.17)	Heng Li *et al.*	https://github.com/lh3/bwa
compleasm (v0.2.7)	Neng Huang *et al.*	https://github.com/huangnengCSU/compleasm
GenomeScope (v2.0)	Rhyker Ranallo-Benavidez *et al.*	https://github.com/schatzlab/genomescope
IGV (v2.18.2)	Robinson *et al.*	https://igv.org/
mashmap (v3.1.1)	Chirag Jain *et al.*	https://github.com/marbl/MashMap
merqury (v1.4.1)	Arang Rhie *et al.*	https://github.com/marbl/merqury
meryl (v1.4.1)	Arang Rhie *et al.*	https://github.com/marbl/merqury
Minimap2 (v2.30)	Heng Li	https://lh3.github.io/minimap2/
ModDotPlot (v0.9.0)	Sweeten *et al.*	https://github.com/marbl/ModDotPlot
paftools	Heng Li	https://lh3.github.io/minimap2/
Picard (v3.3.0)		https://github.com/lh3/bwa
Rustybam (v0.1.33)	Mitchell R Vollger *et al.*	https://www.vollgerlab.com/rustybam/
Samblaster (v0.1.26)	Gregory G. Faust *et al.*	https://github.com/GregoryFaust/samblaster
Samtools (v1.21)	Heng Li *et al.*	https://www.htslib.org/
scipy (v1.13.1)	Virtanen, P., *et al.*	https://scipy.org/
seqtk (v1.4)	Heng Li	https://github.com/lh3/seqtk
statsmodels (v0.14.5)	Seabold and Skipper *et al.*	https://www.statsmodels.org/stable/index.html
Verkko (v2.2.1)	Dmitry Antipov *et al.*	https://github.com/marbl/verkko
Yak (yak-0.1 (r56))	Heng Li	https://github.com/lh3/yak
