# Supplementary material for: Biobank-scale genotyping of Robertsonian translocations reveals hidden structural variation on the human acrocentric chromosomes
Source: bioRxiv. 2026 Mar 27:2026.03.08.710242. Preprint. [Version 2] doi: 10.64898/2026.03.08.710242 (PMC13060921; doi:10.64898/2026.03.08.710242)
Supplement: Supplement 2 [file media-2.pdf]

Supplementary Figures

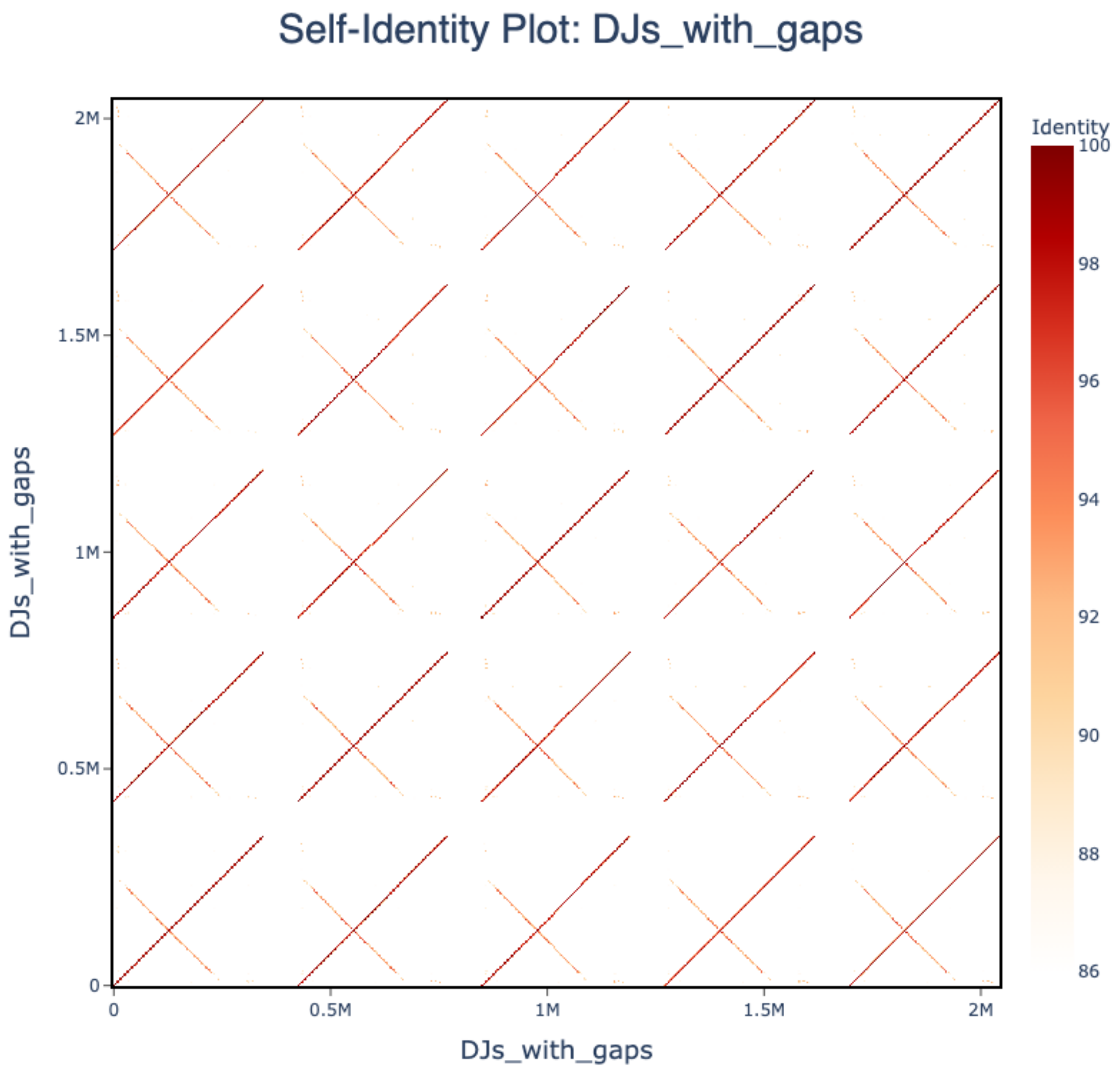

Figure S1. Self-identity dotplot drawn with ModDotplot v0.9.0.

All DJ units on the five acrocentric chromosomes were compared through a self-identity plot. Each DJ unit was separated by an 80 kbp gap for visualization purposes. The DJs are ordered from Chr. 13 to Chr. 22.

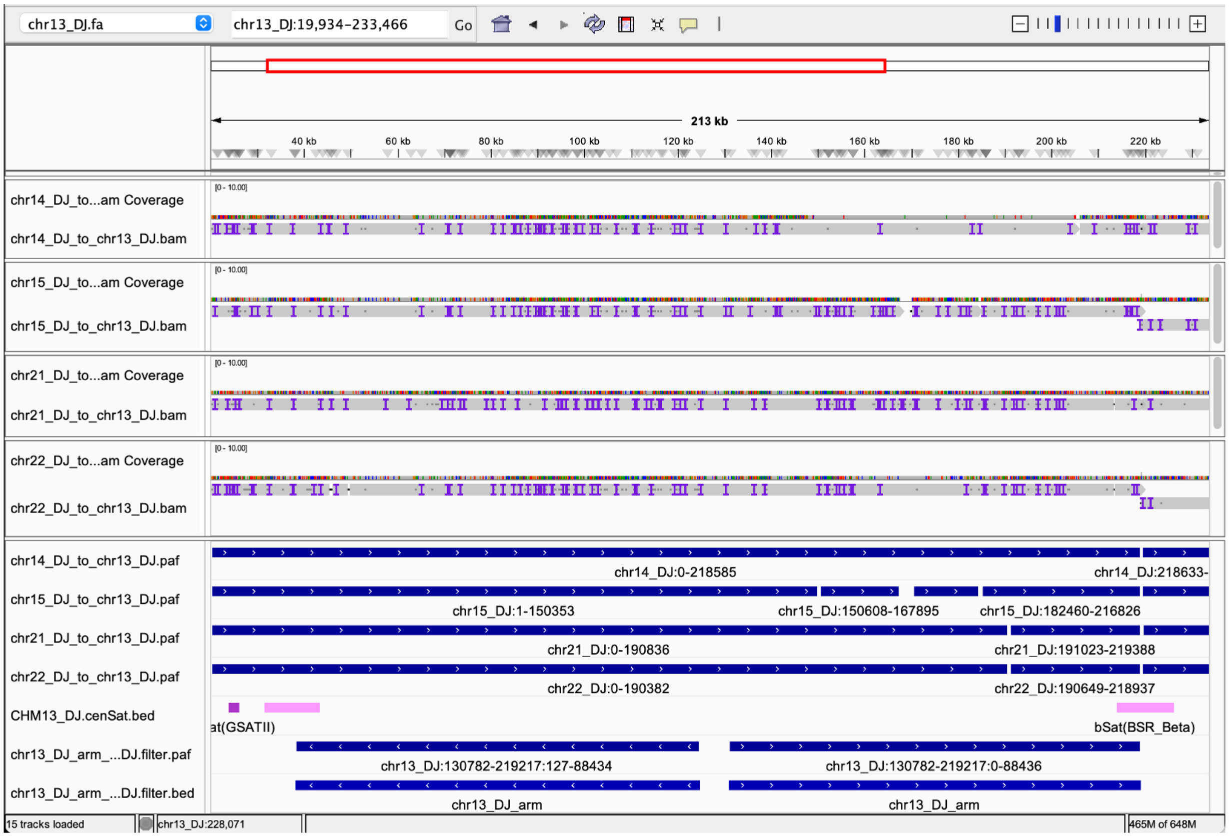

Figure S2. Variations in each DJ unit are enriched at the bSat on the right side, between the DJ arm and DJ flank, and the second DJ arm (right side).

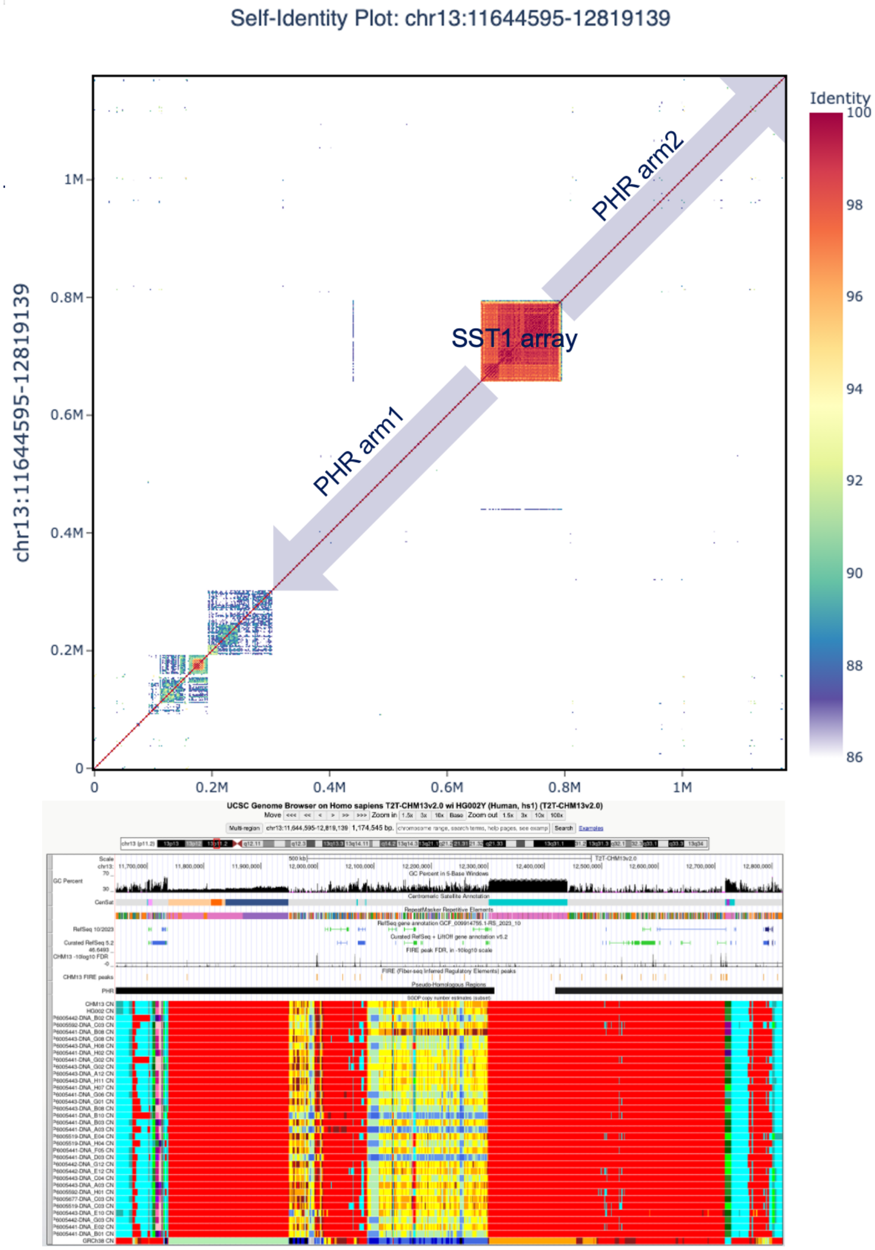

Figure S3. Target PHR region in CHM13.

The target was chosen for the longest form as present on Chr. 13. The PHR showed inconsistent copy numbers from the SGDP copy number variation panel, thus we tried 3 regions - the entire PHR, PHR arm1 and PHR arm2 as shown in the plot. After evaluating the distributions, we chose PHR arm1 as the target for PHR.

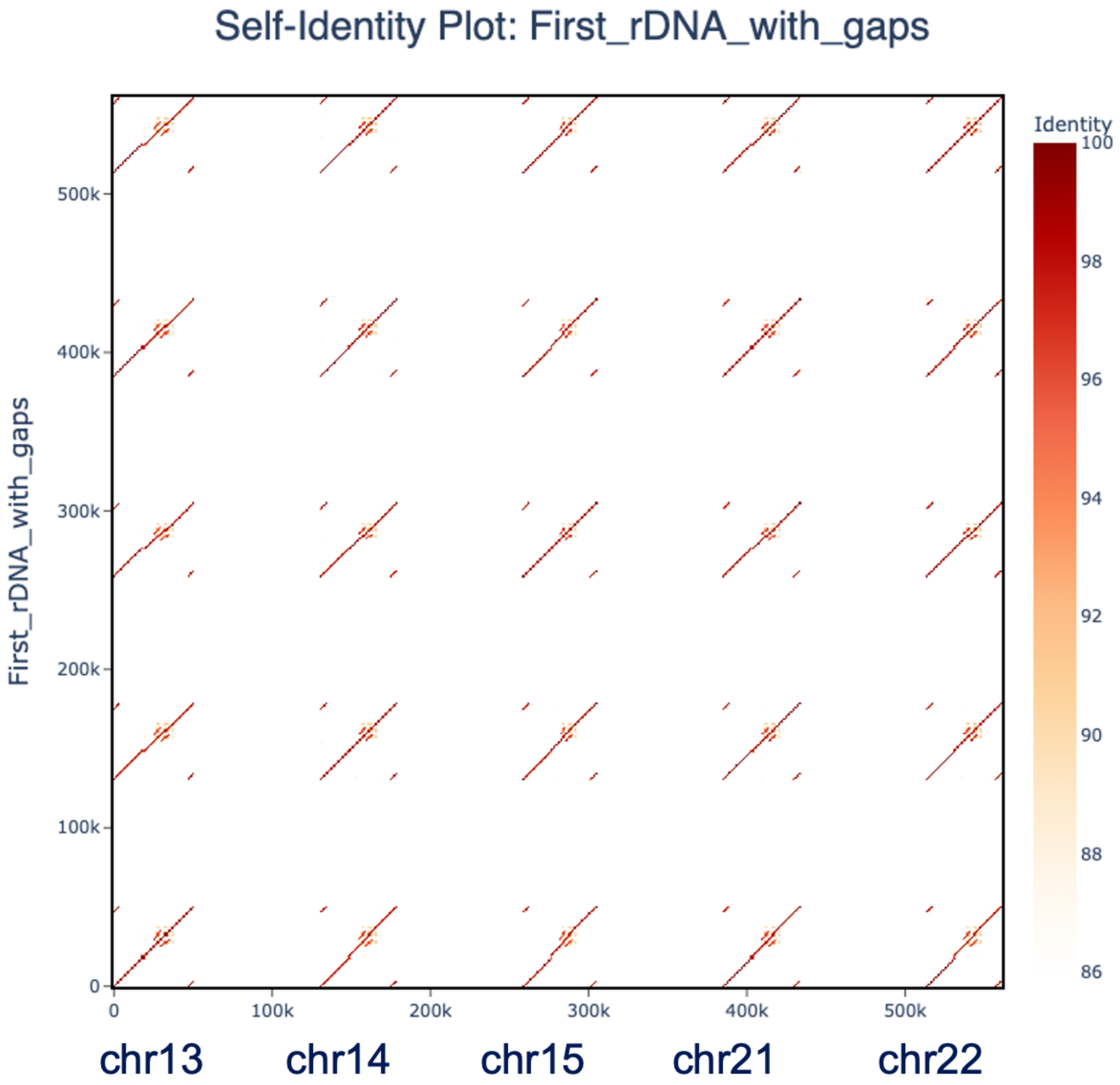

Figure S4. Self-Identity plot for the first units of the rDNAs in each chromosome.

The unit in Chr. 13 had the longest form of rDNA.

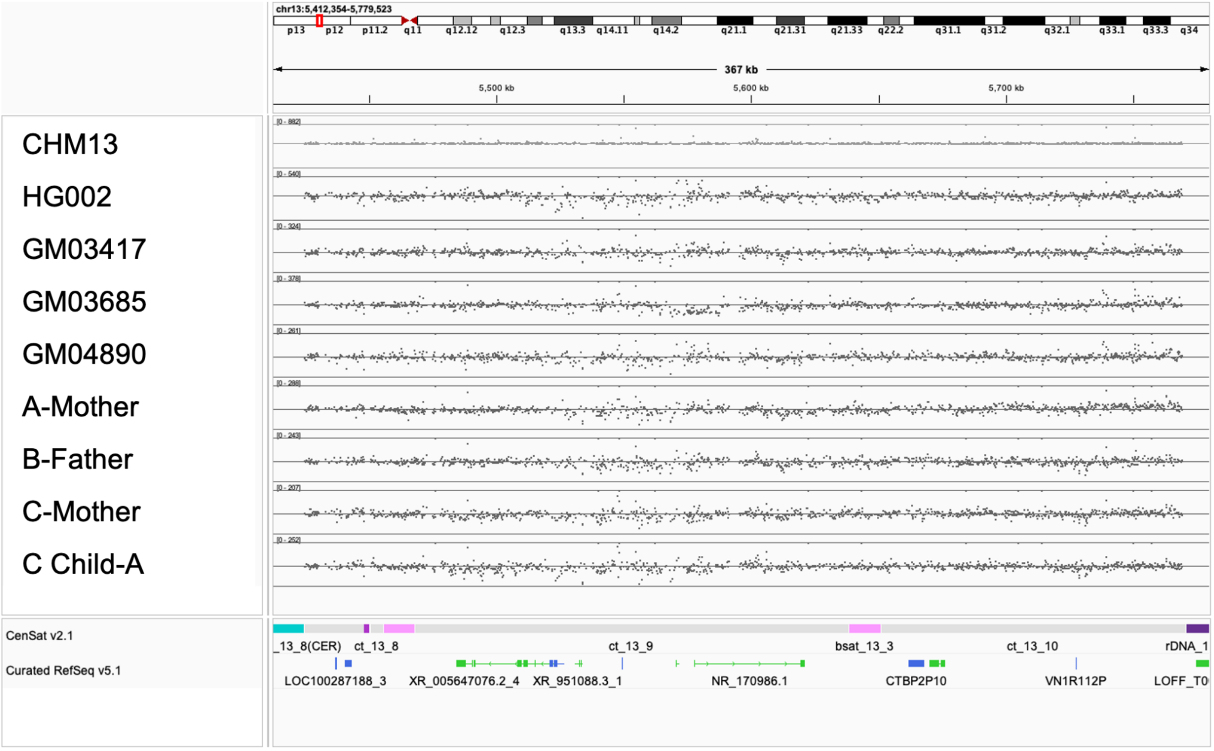

Figure S5. DJ target k-mers in the control samples and the 4 predicted ROB samples found in the RPC.

The k-mer multiplicity is shown as found in the read set on the position of the k-mer as it appears on CHM13 Chr. 13 DJ region. The line across indicates the median of the DJ target k-mer multiplicity. It matched the estimated 10 copies for CHM13 and HG002 and 8 copies for the ROB samples based on the 2 copy peak observed in the k-mer histogram.

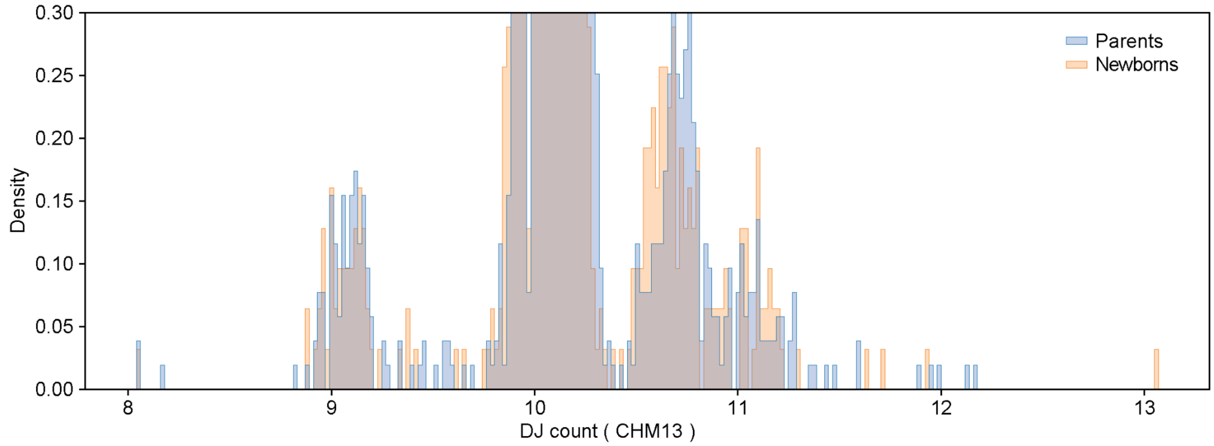

Figure S6. Zoomed-in density distribution of RPC samples: parents vs. newborns.

This panel shows a magnified view of the top panel in Figure 3B.

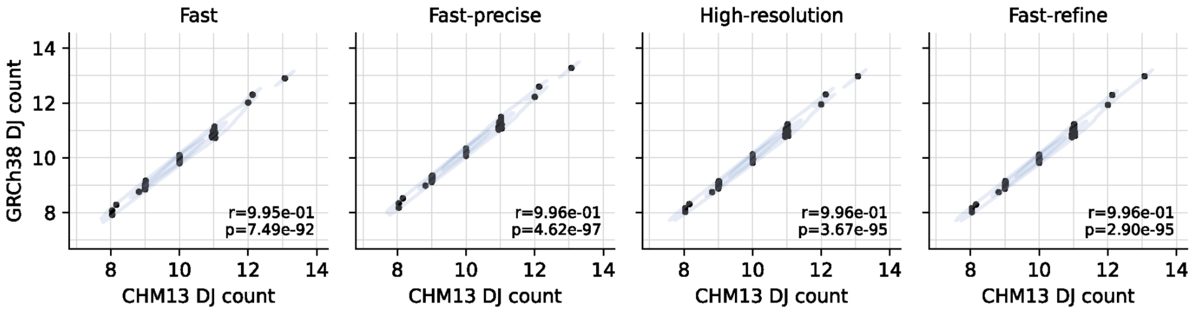

Figure S7. Correlation of DJ copy number estimates between GRCh38 mapping–based methods and the CHM13 mapping–based method.

Pearson correlation analysis was performed between DJ copy number estimates derived from four GRCh38 mapping–based methods and those obtained using the CHM13 mapping–based method.

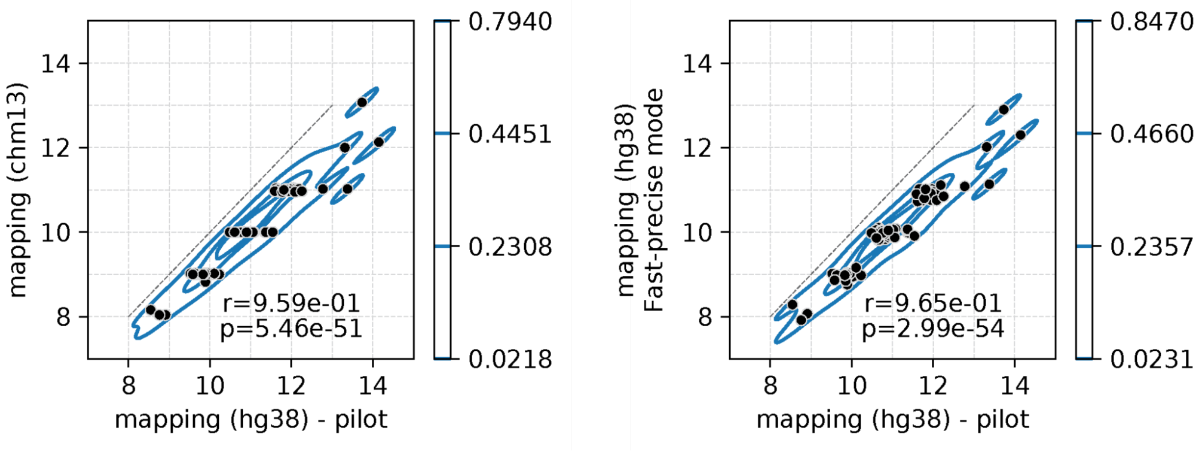

Figure S8. Comparison of mapping approaches using 92 subsampled RPC samples.

Scatter plots comparing pilot-Fast mode with CHM13 mapping, and Fast mode with pilot-Fast mode. Correlations were estimated using Pearson's correlation coefficient.

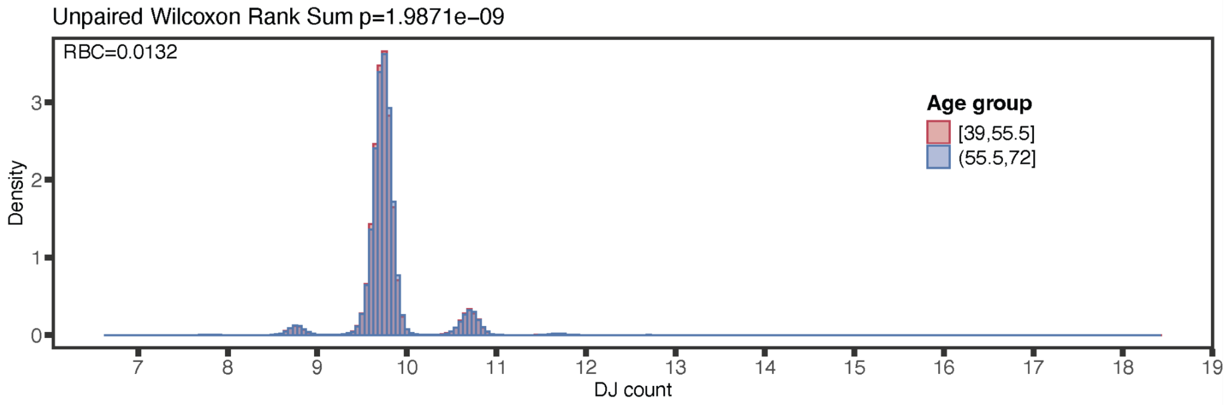

Figure S9. Density plot of DJ by two age groups in the UK Biobank.

The two groups were split at 55.5 years. The results of the unpaired Wilcoxon Rank Sum test are shown. In the RBC (p = 0.0132), the older group tended to have higher DJ counts than the younger group.

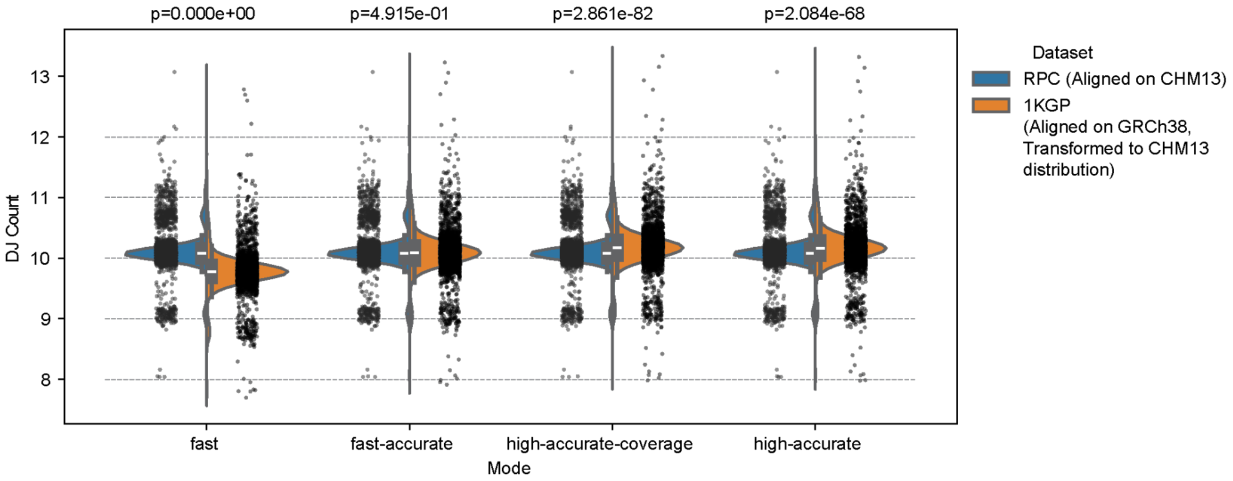

Figure S10. Transform_other_mode. Comparison of DJ copy number distributions between the RPC cohort aligned to CHM13 and the transformed DJ copy numbers from the 1KGP cohort using the corresponding model.

P-values were calculated using the Mann–Whitney U test.

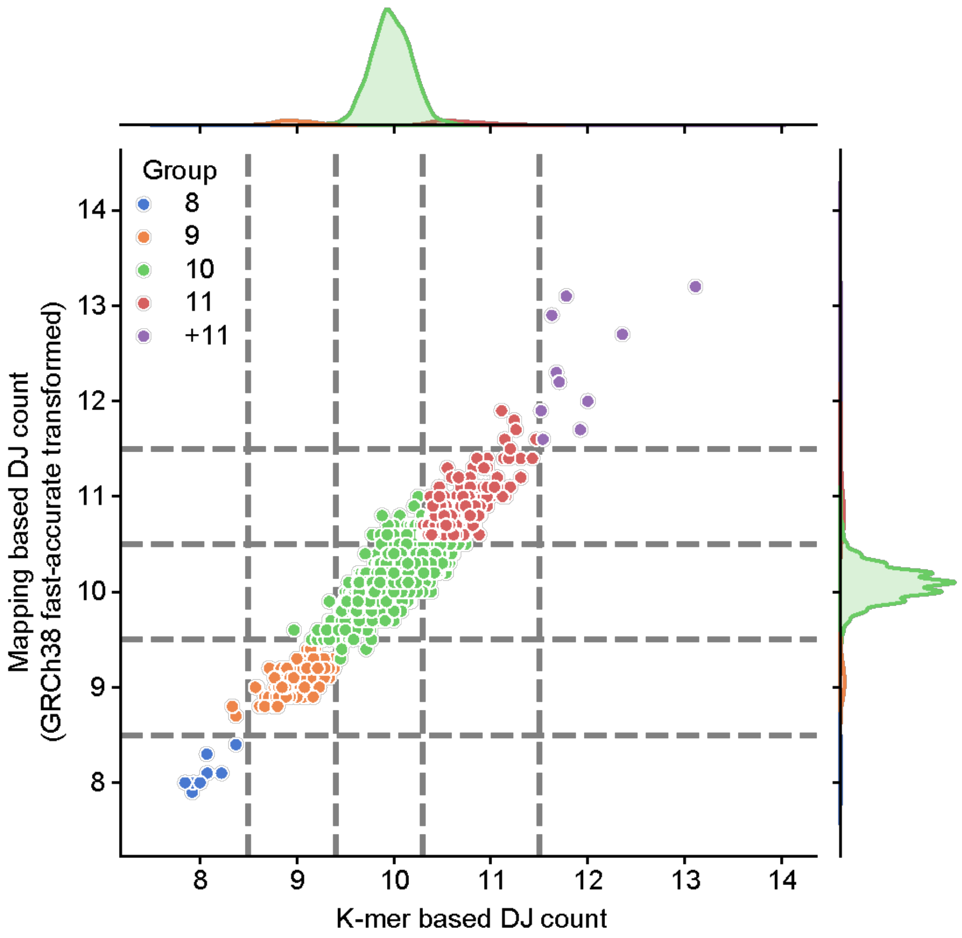

Figure S11. Grouping_1kgp_by_DJ. Grouping of 1KGP samples by DJ values.

Scatter plot showing DJ values for 1KGP samples calculated using the k-mer–based method (x-axis) and the GRCh38 Fast-precise mode (transformed; y-axis). Samples are colored according to group.

CORIELL INSTITUTE
FOR MEDICAL RESEARCH

Cell Line ID: PG01204*0

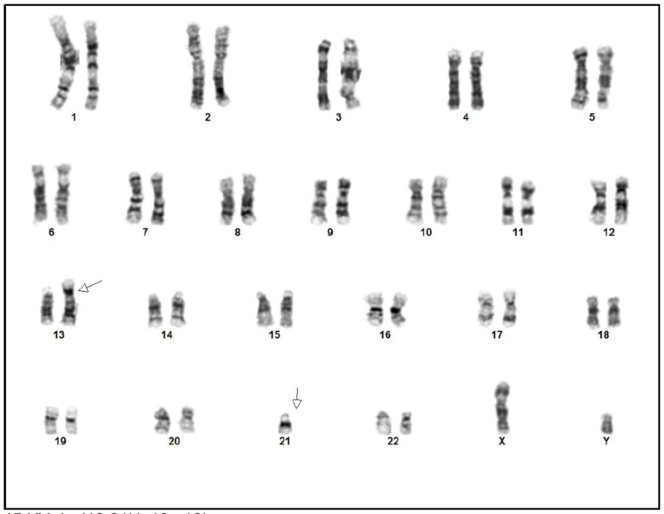

45,XY,der(13;21)(q10;q10)

Cell Line ID: PG01204*0

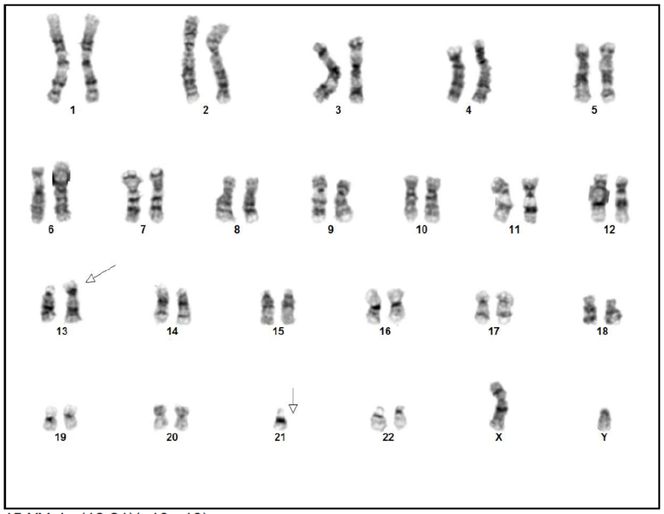

45,XY,der(13;21)(q10;q10)

Form 0600-14 Rev F-110917

403 Haddon Avenue, Camden, NJ 08103-1505 | (856) 966-7377 TEL | (856) 964-0254 FAX | cytogenetics@coriell.org

Figure S12. G-banding report from Coriell. G-banding was performed for HG011204 (Coriell case id: PG01204*0) at passage 6 on Mar. 20, 2024. Cell counts were 20, 5 cells were analyzed and karyotyped.

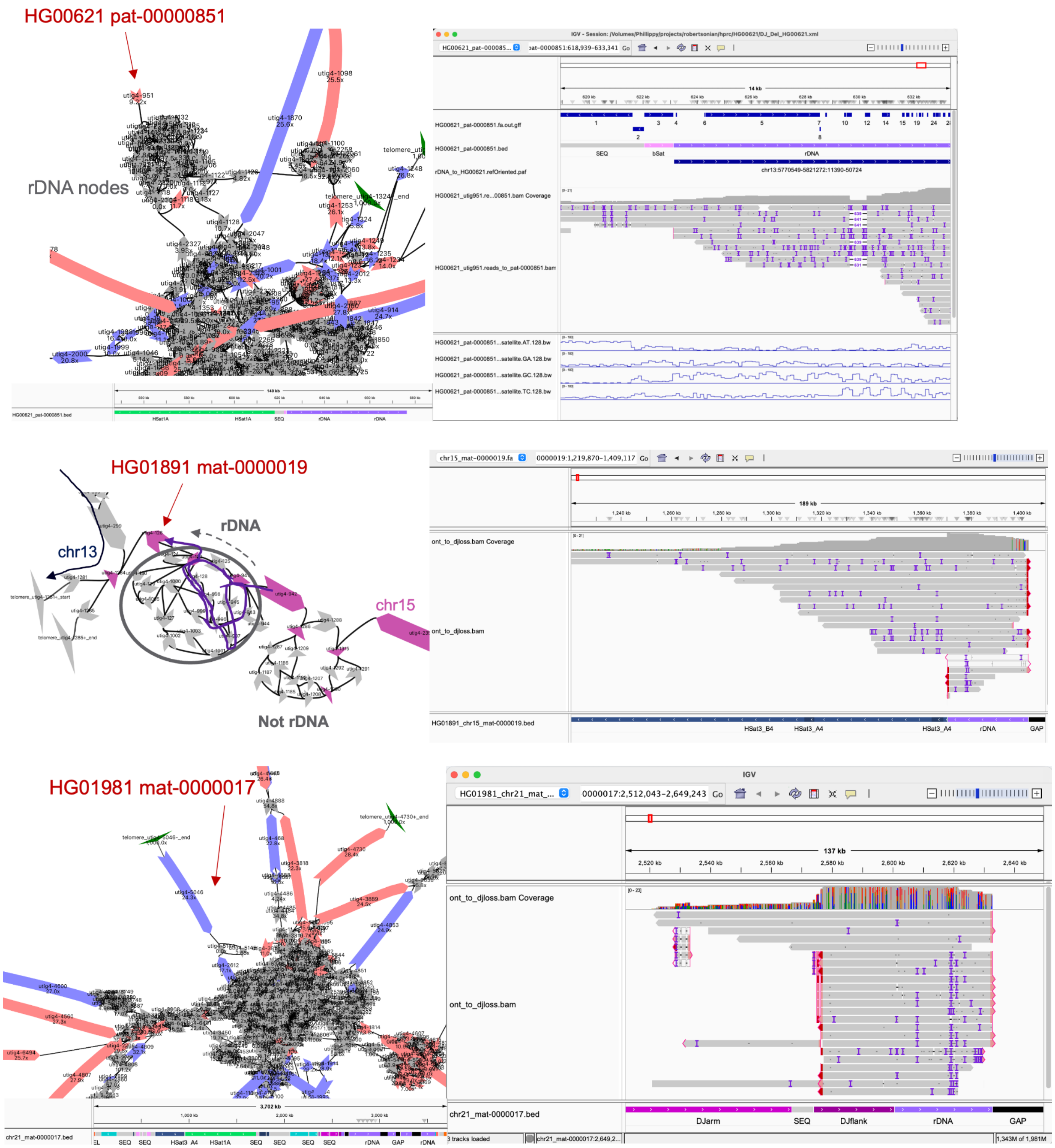

Figure S13. Nodes with truncated or missing DJs on the assembly graph and reads supporting the junction.

The pointed nodes correspond to the sequence shown in **Fig. 5E**, connected to the rDNA node cluster.

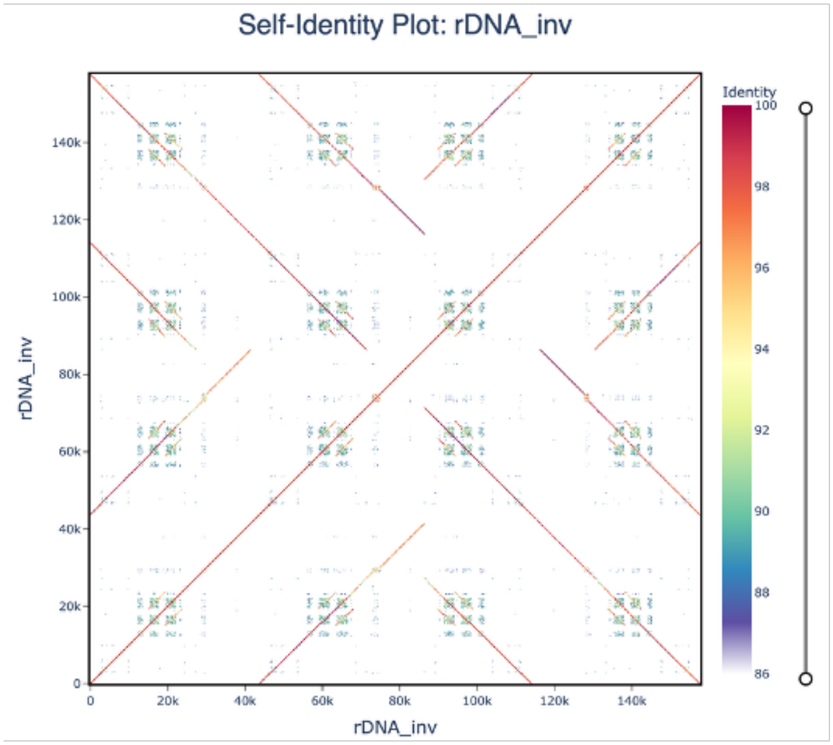

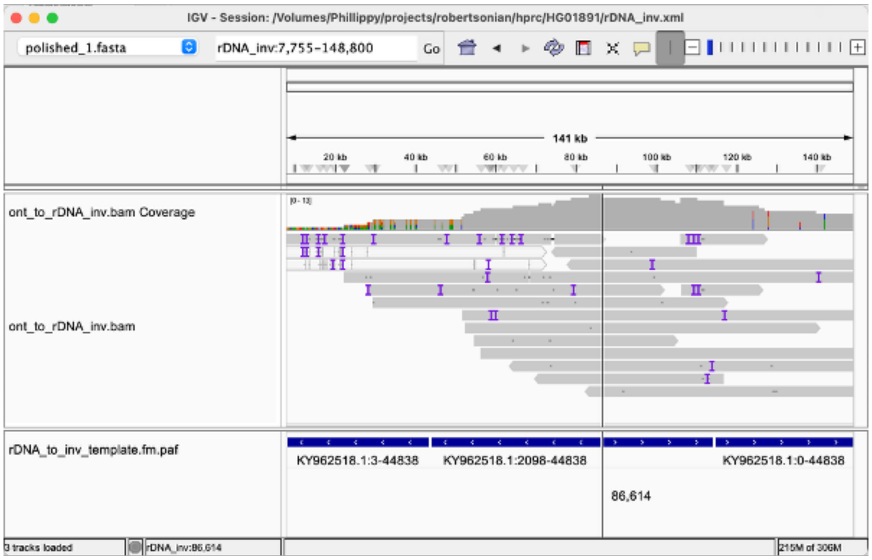

Figure S14. Inverted rDNA array found in HG01891.

ONT-UL reads forming the inverted repeat were extracted and mapped to the rDNA consensus KY962518.1. A template has been constructed based on the read alignments, and polished with Flye v2.9.5. The self-identity plot on the top shows the inverted rDNA junction. Below shows the read alignments at the inversion breakpoint (vertical line).

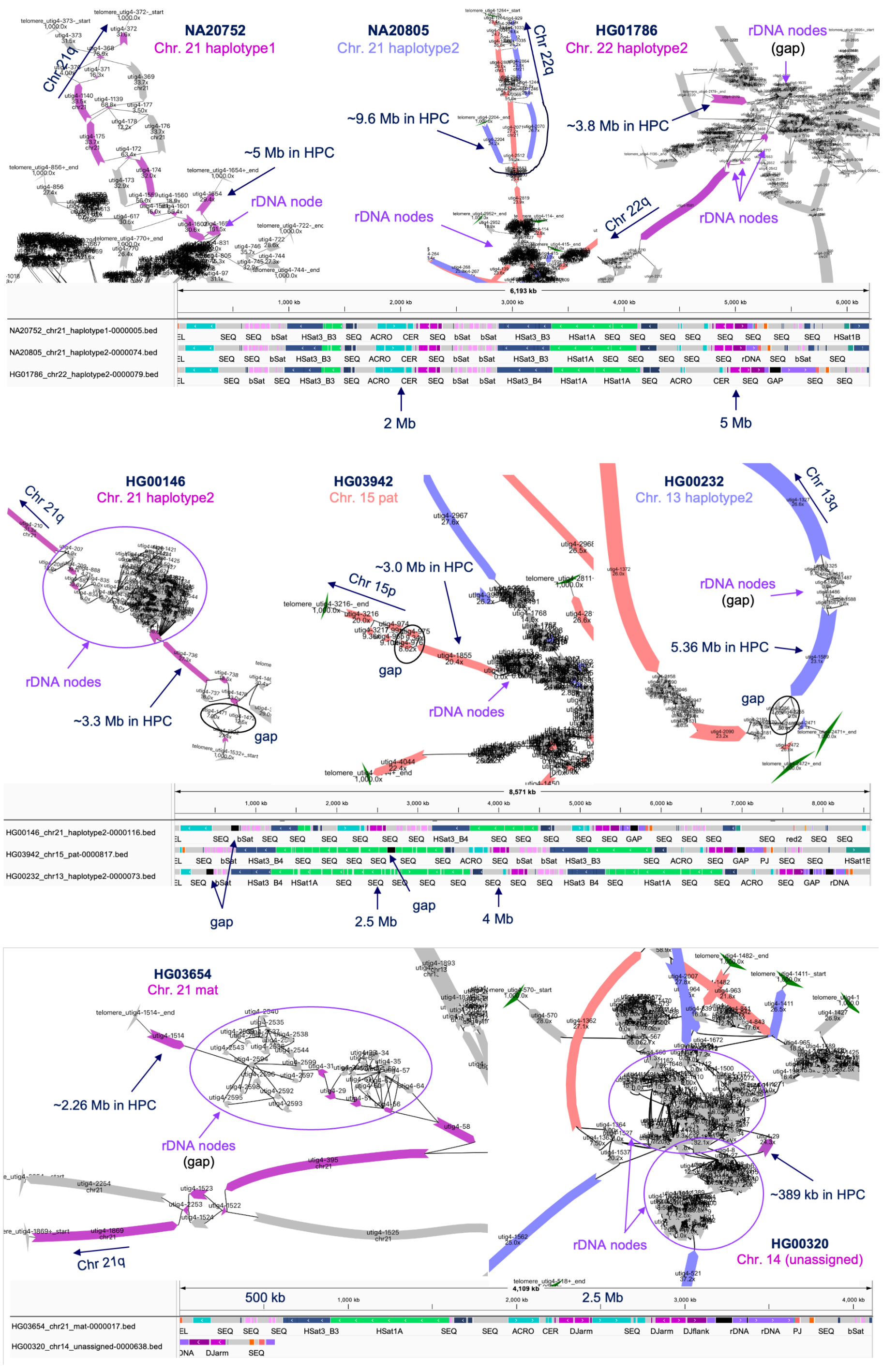

Figure S15. Nodes with the additional DJ copy on the assembly graph.

All duplicated DJ blocks shown in Fig. 5G are found on a single node (pointed in black arrow) in the Verkko assembly graph, indicating it was unambiguously resolved. The node size is shown in homopolymer compressed space (HPC), which is usually smaller than the final consensus. The IGV screen shot shows the consensus repeat annotation with the size in uncompressed space.

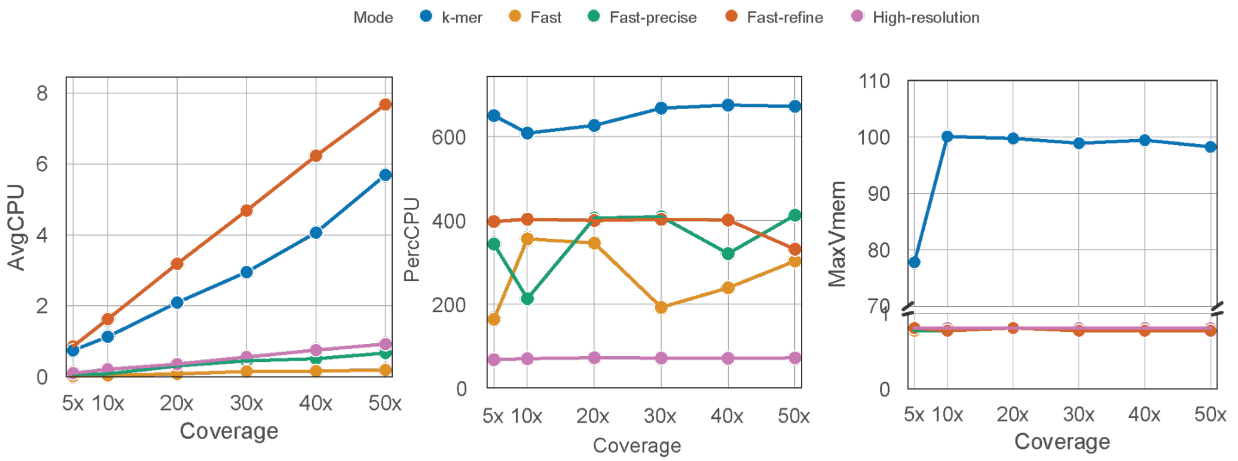

Figure S16. Computational resource usage for each DJ calculation method.

AvgCPU (average CPU time per record in hours), %CPU (average CPU utilization relative to a single core, where 100% equals one fully utilized core) and MaxVmem (maximum virtual memory usage in GB).

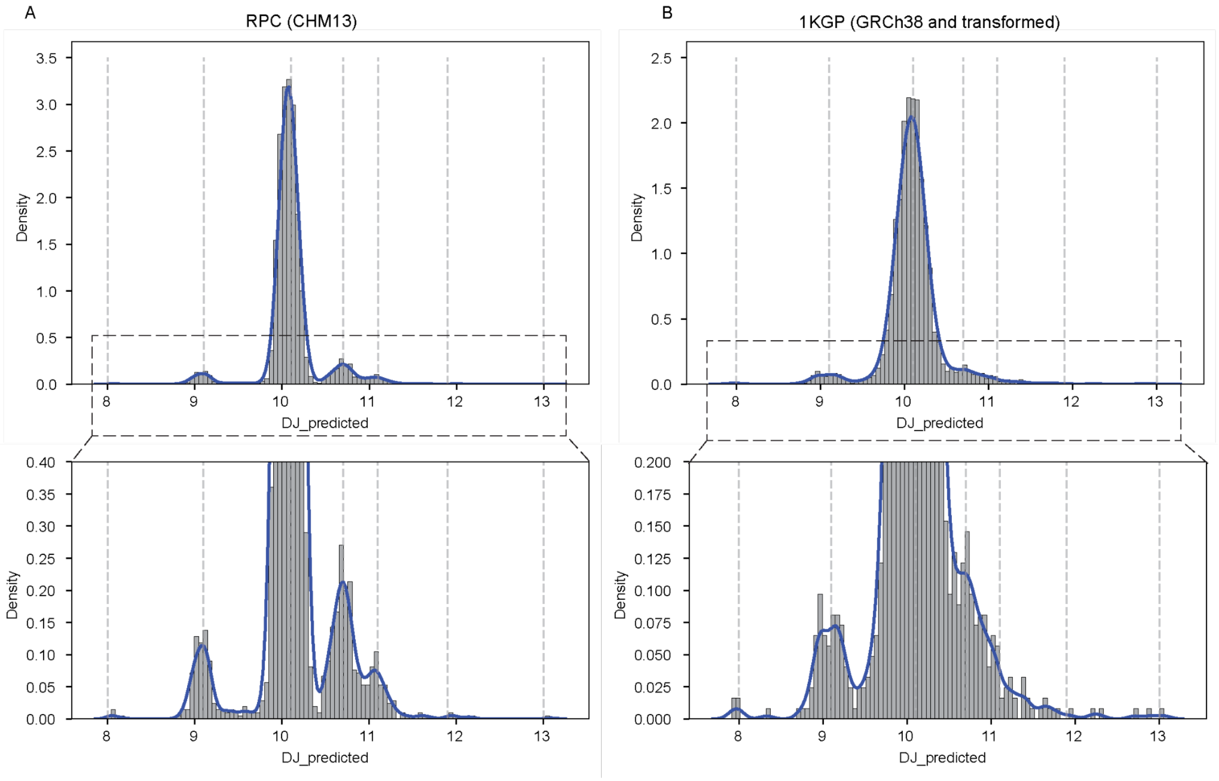

Figure S17. Density and kernel plots of DJ copy number distributions in the RPC cohort (CHM13-aligned) and the 1KGP cohort transformed using the Fast-precise model.
Dotted vertical lines indicate the fixed component means used for Gaussian mixture model (GMM) fitting.

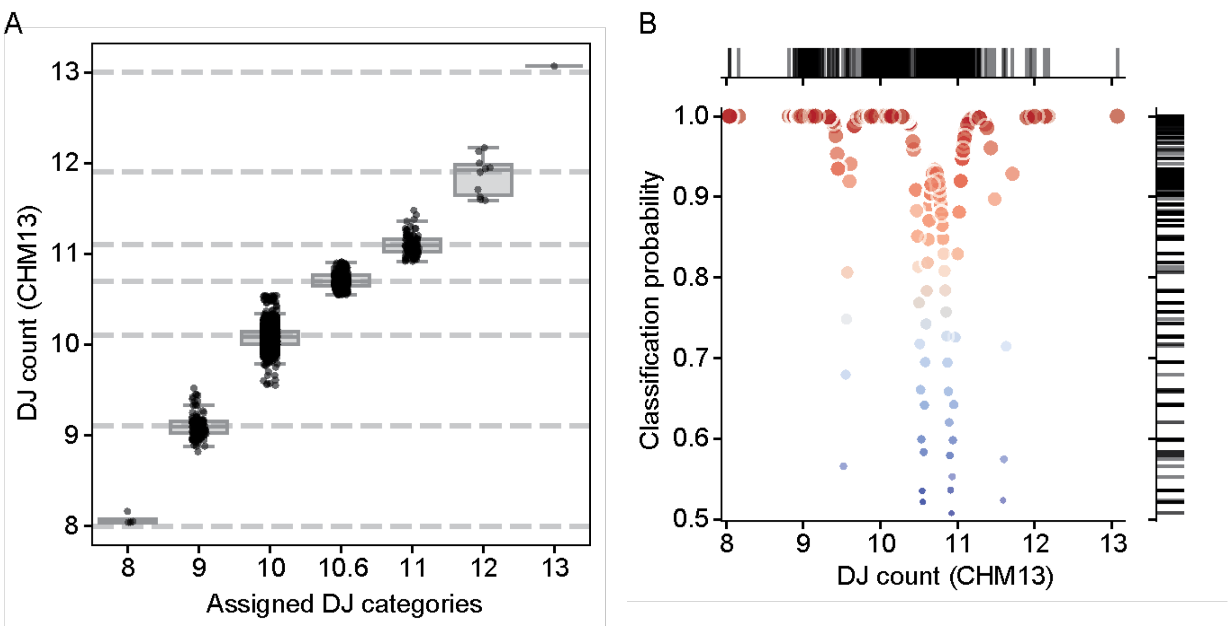

Figure S18. Classification results for the RPC cohort (CHM13-aligned) using a Gaussian mixture model (GMM) trained on DJ copy numbers from the 1KGP cohort (GRCh38-aligned) transformed with the Fast-precise model.

(A) DJ copy numbers derived from CHM13 mapping, grouped by each sample's assigned classification bin. (B) DJ copy numbers and corresponding classification probabilities. Dot color and size represent classification probability, with redder and larger dots indicating higher probability.
